# Design and Synthesis of New Acyl Urea Analogs as Potential σ1R Ligands

**DOI:** 10.3390/molecules28052319

**Published:** 2023-03-02

**Authors:** Rajesh Thapa, Rafael Flores, Kwan H. Cheng, Bereket Mochona, Donald Sikazwe

**Affiliations:** 1 Pharmaceutical Sciences Department, Feik School of Pharmacy, University of the Incarnate Word, San Antonio, TX 78209, USA; 2 Department of Physics and Astronomy and Neuroscience Program, Trinity University, San Antonio, TX 78212, USA; 3 Department of Chemistry, Florida A&M University, Tallahassee, FL 32307, USA

**Keywords:** isoxazole, acyl-urea, modelling, drug-likeness, conformers

## Abstract

In search of synthetically accessible open-ring analogs of PD144418 or 5-(1-propyl-1,2,5,6-tetrahydropyridin-3-yl)-3-(p-tolyl)isoxazole, a highly potent sigma-1 receptor (σ1R) ligand, we herein report the design and synthesis of sixteen arylated acyl urea derivatives. Design aspects included modeling the target compounds for drug-likeness, docking at σ1R crystal structure 5HK1, and contrasting the lower energy molecular conformers with that of the receptor-embedded PD144418—a molecule we opined that our compounds could mimic pharmacologically. Synthesis of our acyl urea target compounds was achieved in two facile steps which involved first generating the *N*-(phenoxycarbonyl) benzamide intermediate and then coupling it with the appropriate amines weakly to strongly nucleophilic amines. Two potential leads (compounds **10** and **12**, with respective in vitro σ1R binding affinities of 2.18 and 9.54 μM) emerged from this series. These leads will undergo further structure optimization with the ultimate goal of developing novel σ1R ligands for testing in neurodegeneration models of Alzheimer’s disease (AD).

## 1. Introduction

**Figure 1:**
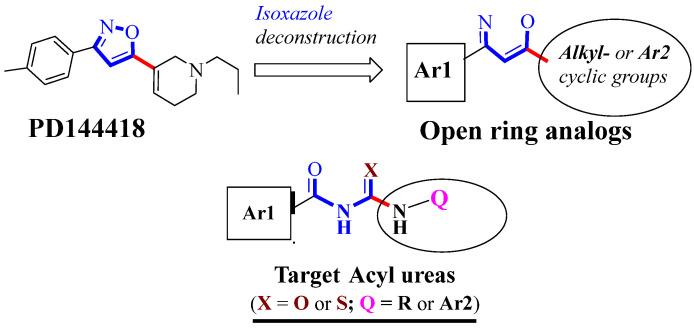
Acyl urea scaffold with alkyl (**R**), aromatic/heteroaromatic features (**Ar1** and **Ar2**).

Inspired by the highly potent σ1R ligand [PD144418 or 5-(1-propyl-1,2,5,6-tetrahydropyridin-3-yl)-3-(p-tolyl)isoxazole] which requires multi-step synthesis, we opted to deconstruct its *isoxazole* ring and convert it into a more readily accessible *acyl urea scaffold.* This structural change facilitates quick synthetic access to a broad range of open-ring acyl-urea analogs, with patentable space, as potential σ1R ligands—evidenced by compound SW-43 [1]. The acyl urea scaffold also allows access to analogs with additional rotatable bonds, conformations, and aqueous solubility due to H-donor/acceptor capabilities [2]. Interestingly, small molecules containing urea pharmacophores have adaptable clinical applications, therefore medicinal chemists are also exploring the utility of this functionality in potential anti-neurodegenerative agents [2,3,4]. In fact, literature not only indicates that σ1R is a promising druggable target in neurodegenerative pathologies like AD, but it also suggests additional roles in other disease pharmacologies (addiction, pain, cancer proliferation, HIV, COVID-19, etc.) [1,5,6,7,8]. Now, the unsatisfactory level of the urea’s blood-brain barrier (BBB) penetration due to H-bonding/ionization/polarity/molecular flexibility [2] suggests making structural elaborations to endow derivatives with “drug-like” pharmacokinetics (absorption, distribution, metabolism, elimination, toxicity or ADMET). Drug-like pharmacokinetics are determined by the molecule’s hydrophobicity, flexibility, size, and electronic nature [9,10,11,12], and computational predictions can help identify such in early drug design. To that end, our sixteen compounds were computationally profiled for pharmacokinetics and docked at σ1R prior to their synthesis. Structural design aspects of our arylated acyl-ureas are illustrated in Figure 1.

Online Molinspiration [13] and Osiris property explorer [14] were used to calculate/predict drug-like molecular properties for the target compounds. These studies involved retrieving the σ1R crystal structure (5HK1) with embedded ligand (PD144418), preparation (e.g., H_2_O and ligand removal), and docking the target molecules via the PyRx and AutoDock Vina suite [15,16]. All sixteen compounds comparably docked to the 5HK1 ligand binding site, suggesting that acyl ureas could also bind to σ1R. Amino acid (AA) interactions in the receptor binding site were also revealed. Synthetically, a variety of approaches for obtaining acyl ureas were reviewed [17,18,19,20,21,22,23,24,25,26,27,28,29] and the most feasible conditions were attempted with limited success—our goal was to obtain our compounds using the shortest and most cost-effective approach. In our hands, even the reported convenient Stokes’ method [23] which required obtaining the same key *N-*(phenoxycabonyl)benzamide intermediate from diphenyl carbonate, proved futile. Instead, ^1^H NMR analysis of reaction mixtures revealed phenol, methyl benzoate, and remaining starting materials. We therefore slightly modified Stokes’ conditions [23] by preparing the *N-*(phenoxycabonyl)benzamide intermediate from phenol and benzoyl isocynate. Coupling the intermediate with amines or amides or thioamides via simple nucleophilic displacement afforded our series of *N*-acyl ureas plus *N*-acyl thioureas (Table 1) in good yields (>64%). To confirm our pharmacological assumptions regarding these molecules behaving like PD144418, the compounds were sent to the National Institute of Mental Health’s Psychoactive Drug Screening Program and evaluated in radioligand displacement assays [30] to determine their in vitro σ1R affinities or K_i_ values.

## 2. Results and Discussion

### 2.1. Chemistry

**Scheme 1:**
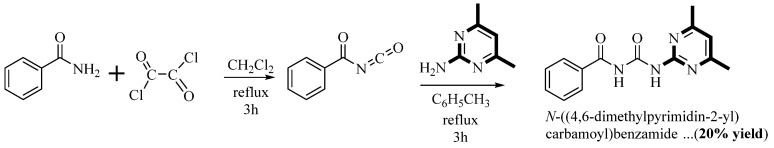
Classical synthesis of acyl ureas from benzamides.

Literature is replete with synthetic options for obtaining acyl ureas. For this project, we reviewed and tried several established approaches [17,18,19,20,21,22,23,24,25,26,27,28,29]. However, our attempts at synthesizing the target compounds were besieged by complex reaction mixtures and poor reaction yields after tedious purifications. For instance, classical conditions (e.g., Scheme 1) which involve initially reacting benzamides and reactive oxalyl chloride followed by the addition of corresponding amines, lead us to dark reaction mixtures with multiple product spots by thin layer chromatography (TLC). Perhaps the acidic conditions were too reactive or unstable and therefore thwarted desired product formation.

**Scheme 2:**
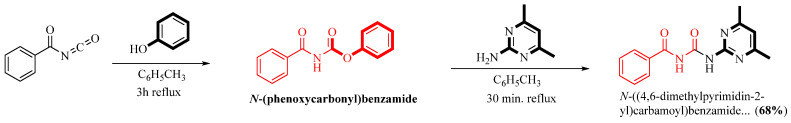
Two-step synthesis of acyl ureas from isocynates and phenol via the *N*-(phenoxycarbonyl)benzamide intermediate.

We eventually settled on the reported convenient Stokes’ method [23] and slightly modified it by first generating the *N*-(phenoxycarbonyl)benzamide intermediate from benzoyl isocyanate and phenol (rather than from diphenylcarbonate). Obtaining this key intermediate, in this manner, facilitated the synthesis of our target acyl urea or *N*-benzoyl urea compounds in Table 1 conveniently and cost-effectively, in two steps (Scheme 2) and in good yields (64–94%). Essentially, our compounds were synthesized by directly coupling amines or amides, or thioamides with a readily scalable *N*-(phenoxycarbonyl)benzamide intermediate, under reflux, via simple nucleophilic displacements.

This synthetic approach tolerated both weakly and strongly nucleophilic amines, under mild reaction conditions, and is amenable to both parallel and high throughput access to diverse arylated acyl ureas for different applications. All compounds were purified (via filtration and/or combi-Flash) and structurally confirmed by spectroscopic methods [^1^H and ^13^C-NMR, high-resolution MS (ES)]. ^1^H NMRs of this family of compounds showed the presence of an exchangeable signal characterizing the -NH proton at the region δ 9 to 15 ppm DMSO-*d*_6_. ^13^C NMRs ascertained the presence of two to three C=O groups.

### 2.2. Drug-Likeness Predictions

At a minimum, drug-likeness implies ligands exhibiting Lipinski’s *rule of five* (Ro5), topological polar surface area (TPSA), and the number of rotatable bond (NRB) limit considerations [9,10,11,12,31]. Table 2 itemizes partial molecular descriptors and drug-likeness properties obtained via Molinspiration /Osiris [13,14] predictions on our target compounds (entries 1–16). Ro5 upper limits are listed in the bottom row.

All target compounds were of low molecular weight (MW < 500), their range was 231.21–396.44. Low molecular weight drug molecules are readily absorbed, diffuse, and are easier to transport versus heavier molecules. Lipophilicity (logP) and TPSA values are predictors of drug oral bioavailability. Calculated log *p* values of 1.53–3.53 were in the acceptable range of drug molecules capable of penetrating biological membranes including the blood-brain barrier (BBB). The numbers of H-bond acceptors (O and N atoms) and H-bond donors (OH and NH) in the target compounds were within the Ro5 guidelines. TPSA values for all molecules were within the acceptable range. TPSA values are used to characterize potential drug absorption (including intestinal absorption), bioavailability, Caco-2 permeability, and BBB penetration. These values are calculated from the surface areas occupied by oxygen, nitrogen, and the hydrogen atoms attached to them. Thus, the TPSA is closely related to the hydrogen bonding potential of a compound. Interestingly, all compounds exhibited 52.65–119.7 Å TPSA values, indicating potential good oral bioavailabilities. Good bioavailability is more likely for compounds with ≤10 rotatable bonds and TPSA of ≤140 Å. As the NRBs increase, the molecule becomes more flexible and more adaptable for efficient interactions in particular binding pockets. Interestingly all compounds have 1- 4 rotatable bonds and are flexible. Positive drug-likeness scores suggest that the designed molecules retain predominant structural features of known/common commercial drugs. All but two of the sixteen target molecules (compounds **2** and **9**) exhibited positive drug-likeness scores.

### 2.3. Molecular Docking

**Figure 2:**
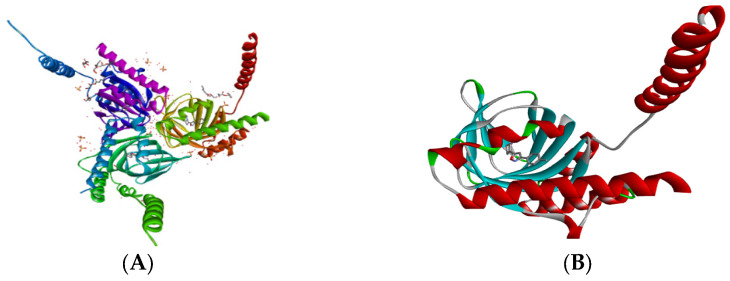
Schematic structure of the (**A**) X-ray crystallographic structure of σ1R co-crystallized with PD14418 (PDB:5HK1) (**B**) Discovery Studio prepared 5HK1.

Docking was conducted to evaluate protein-ligand interactions and predict binding affinities of the target compounds versus the comparator potent σ1R ligand PD144418. Our target molecules were docked using the PyRx and AutoDock Vina platforms [15,16]. Analysis of the docking scores and prediction of binding modes between the ligand and σ1R was conducted using BIOVIA Discovery Studio 4.5 visualizer [32]. Figure 2 contrasts the before (A) and after (B) preparation visualizations of PD14418 in σ1R or 5HK1 binding pocket.

Docking yielded the most stable conformer free-binding energy scores (i.e., binding affinities in kcal/mol) and H-bonding interactions within the active site of σ1R (Table 3).

**Figure 3:**
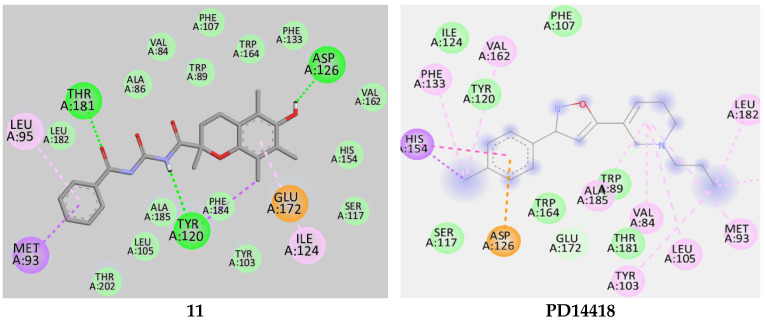
2D Interactions of representative compound **11** and **PD14418** with 5HK1. Legend: (Dark green, Conventional H-bonding, Light green (π-donor hydrogen), Purple (π-sigma), Pink (π-alkyl), and Orange (π-anion) polar and non-polar interactions.

Additionally, docking revealed relevant amino acids for bonding interactions in the active site of σ1R (5HK1). Example interactions are illustrated in Figure 3.

The binding affinity values of the most stable/best-docked 5HK1 bound conformers ranged from −11.40 to −8.6 kcal/mol. The binding energies of all compounds were significant and comparable to the reference ligand PD14418 (−10.2 kcal/mol) and were commonly due to conventional H-bonding interactions between GLU 172 and the amide bond hydrogen atom. Representative compound **11** exhibited the *strongest* (three conventional hydrogen bonds via -OH, carbonyl, and -NH) *polar* interactions with ASP126, THR 181, and TYR 120 residues. In addition to the H-bond interactions illustrated in Figure 3, the 5HK1-compound **11** complexes were also stabilized by π-sigma (Tyr 120) and π-anion (Glu 172, and Asp 126) interactions which also contributed to the calculated lowest root mean square deviation (RMSD) value and provided the best-fit result. On the other hand, the comparator (PD14418) formed: π-sigma with His 194; π-alkyl with Val 84, Leu 105, Phe 133, Val 162, and Ala 185; π-anion with Asp 126; π-donor with Phe 107, Ser 117, Tyr 120, Ile 124, Trp 164, Glu 172 and Thr 181residues.

Computational (kcal/mol) versus in vitro (μM, from the National Institute of Mental Health Psychoactive Drug Screening Program or NIMH-PDSP [https://pdsp.unc.edu/pdspweb/ (accessed 28 February 2023)] σ1R binding affinity data are illustrated in Table 4.

It is worth noting that although we used modeling/docking to guide our synthetic target prioritization, we did not observe a good correlation between predicted versus the in vitro affinity data. This discrepancy is perhaps a result of quantitative limitations of the free and open-source software (FOSS) platforms (e.g., Open Babel) we utilized. For example, ligand protonation states can affect virtual binding affinities [34], ergo, multiple ionization states needed to be included in the prediction data. Moving forward, we endeavor to utilize more robust computational platforms (either through collaborations or by subscription) to optimize the binding affinities of the two leads before synthesizing any follow-up compounds for the SAR studies.

## 3. Experimental

### 3.1. Materials and General Methods

Reagents and solvents were purchased from vendors [Fischer Scientific and Acros Organics (Waltham, MA, USA)/Sigma Aldrich (St. Louis, MO, USA] and used as received unless otherwise indicated. All reactions were performed using established procedures. Melting points were taken on the Mel-temp capillary apparatus and reported uncorrected. Teledyne Combiflash Rf flash chromatography fitted with Redisep Rf silica gel cartridges was used for compound purifications. Analytical TLC plates from EM Science (silica Gel 60 F_254_) were used. ^1^H and ^13^C spectra (Supplementary Materials) were recorded using CDCl_3_ or DMSO-d_6_ as solvents on a Bruker 300 MHz spectrometer at ambient probe temperature unless otherwise indicated. ^1^H and ^13^C chemical shifts are reported versus SiMe_4_ and were determined by reference to the residual ^1^H and ^13^C solvent peaks. Coupling constants (*J*) are reported in hertz (Hz). Characterization data were reported as follows: chemical shift, multiplicity (s = singlet, d = doublet, t = triplet, q = quartet, br = broad, m = multiplet), coupling constants, number of protons, and mass-to-charge (m/z) ratios. High-Resolution Mass Spectrometry (HRMS) was carried out using an Agilent 6230 electrospray ionization time-of-flight instrument. Where necessary, the purity/mass of the target compounds was determined by analytical reversed-phase high-performance liquid chromatography/mass spectrometer (HPLC/MS) tandem on a Dionex Ultimate 3000 HPLC system. HPLC was conducted using Nova-Pak C18 Column, 60Å, 4 µm, (4.6 mm × 150 mm) at ambient temperature, and a flow rate of 1.0 mL/min., CH_3_CN & H_2_O eluent (containing 0.1% acetic acid); gradient, 5% CH_3_CN to 100% CH_3_CN; 8 min.; UV detection at 254 nm. Mass was obtained in positive ion mode using heated electrospray ionization (ISQ-HESI) source. MS conditions: capillary voltage, 3.0000 V; drying gas flow, 0.2mL/min; vaporizer gas temperature, 350 °C; and gas pressure, 28.8 psig. MS data were acquired with Chromeleon 7.

### 3.2. Synthetic Procedures and Compound Data

Compounds **1–15**. Individual amines/amides/thioamides were added to *N*-(Phenoxycarbonyl) benzamide—a versatile common precursor in toluene (20 mL), and resulting mixtures were stirred at room temperature (25 °C) for 10 min, followed by 30–60 min of reflux. Suspended solids that formed upon cooling were collected by filtration, triple rinsed (with 0 °C toluene), and dried under vacuum to afford the desired products. In cases where suspensions did not form after cooling, reaction mixtures were concentrated by rotavapor and then cooled to room temperature to promote crystallization or solidification. Flash chromatography was used as needed to further purify the products. This procedure provided acyl urea and acyl thiourea compounds (**1–15**) in 64–94% yields. Compound **16** was prepared in 65% yield by directly condensing benzoyl isothiocyanate with 2-aminoisoquinoline in THF for 2 h.

#### 3.2.1. *N*-((5-Methylisoxazol-3-yl)carbamoyl)benzamide (**1**)

*N*-(phenoxycarbonyl) benzamide (632 mg, 2.62 mmol) was reacted with 3-amino-5-methylisoxazole (290 mg, 2.95 mmol) in toluene (60 min reflux) and the pure product was collected as a white crystalline solid, yield: (490 mg, 76%), m.p. 212–215 °C. ^1^H NMR (300 MHz, CDCl_3_) δ 11.20 (s, 1H, NH), 9.04 (s, 1H, NH), 7.95 (dd, *J* = 7.3, 1.8 Hz, 2H, arom.CH), 7.72–7.60 (m, 3H, arom.CH), 6.59 (s, 1H, arom.CH), 2.43 (d, *J* = 0.9 Hz, 3H, CH_3_). ^13^C NMR (75 MHz, CDCl_3_) δ 170.30, 168.13, 157.71, 151.18, 134.06, 132.05, 129.43, 128.09, 97.01, 13.01. HRMS (ES) m/z calcd. for (C_12_H_11_N_3_O_3_; M + H)^+^ 246.0878 found 246.0874.

#### 3.2.2. *N*-((1H-Benzo[D]imidazol-2-yl)carbamothioyl)benzamide (**2**)

*N*-(phenoxycarbonyl) benzamide (560 mg, 2.32 mmol) was condensed with 5-aminoindole (331 mg, 2.49 mmol) in toluene (30 min reflux). The product was obtained as a white crystalline solid, yield: (615 mg, 94%), m.p. 220–223 °C. ^1^H NMR (300 MHz, DMSO-d_6_) δ 11.10 (s, 1H, NH), 10.99 (s, 1H, NH), 10.77 (s, 1H, NH), 8.10–8.00 (m, 2H, arom.CH), 7.85 (d, *J* = 2.0 Hz, 1H, arom.CH), 7.72–7.60 (m, 1H, arom.CH), 7.61–7.45 (m, 2H, arom.CH), 7.42–7.32 (m, 2H, arom.CH), 7.18 (dd, *J* = 8.7, 2.1 Hz, 1H, arom.CH), 6.42 (ddd, *J* = 3.0, 1.9, 0.9 Hz, 1H, arom.CH). ^13^C NMR (75 MHz, DMSO-d_6_) δ 169.34, 151.78, 133.53, 132.81, 129.91, 129.59, 129.13, 128.59, 128.10, 127.87, 126.71, 115.97, 115.67, 112.11, 112.08, 101.70, 79.38. HRMS (ES) m/z calcd. for (C_16_H_13_N_3_O_2_; M + H)^+^ 280.1086 found 280.1080.

#### 3.2.3. *N*-((Pyridine-2-carbonothioyl)carbamoyl)benzamide (**3**)

*N*-(phenoxycarbonyl) benzamide (336 mg, 1.39 mmol) and 2-pyridine thioamide (210 mg, 1.52 mmol) were reacted in toluene (60 min reflux). The desired product was obtained as a purple solid, yield: (304 mg, 75%), m.p. 180–182 °C. ^1^H NMR (300 MHz, DMSO-d_6_) δ 14.63 (s, 1H, NH), 11.51 (s, 1H, NH), 8.72 (ddd, *J* = 4.7, 1.8, 0.9 Hz, 1H, arom.CH), 8.46 (dt, *J* = 8.1, 1.1 Hz, 1H, arom.CH), 8.12–8.01 (m, 3H, arom.CH), 7.79–7.64 (m, 2H, arom.CH), 7.61–7.52 (m, 2H, arom.CH). ^13^C NMR (75 MHz, DMSO-d_6_) δ 194.24, 168.41, 151.60, 149.75, 147.68, 138.12, 133.32, 132.10, 128.59, 127.47, 124.87. HRMS (ES) m/z calcd. for (C_14_H_11_N_3_O_2_S; M + H)^+^ 286.0650 found 286.0642.

#### 3.2.4. *N*-((2-Methyl-1H-indol-5-yl)carbamoyl)benzamide (**4**)

*N*-(phenoxycarbonyl) benzamide (740 mg, 3.07 mmol) was reacted with 2-amino-5-methylindole (510 mg, 3.48 mmol) in toluene (40 min reflux). The purified product was a dark brown solid, yield: (635 mg, 71%), m.p. 209–211 °C. ^1^H NMR (300 MHz, DMSO-d_6_) δ 10.93 (d, *J* = 15.5 Hz, 2H, NH), 10.73 (s, 1H, NH), 8.09–7.99 (m, 2H, arom.CH), 7.72–7.62 (m, 2H, arom.CH), 7.55 (dd, *J* = 8.2, 6.8 Hz, 2H, arom.CH), 7.23 (d, *J* = 8.6 Hz, 1H, arom.CH), 7.08 (dd, *J* = 8.6, 2.1 Hz, 1H, arom.CH), 6.11 (dt, *J* = 2.0, 1.0 Hz, 1H, arom.CH), 2.41–2.34 (m, 3H, CH_3_). ^13^C NMR (75 MHz, DMSO-d_6_) δ 169.32, 151.71, 137.22, 133.72, 133.49, 132.80, 129.90, 129.39, 129.11, 128.57, 115.66, 114.68, 111.17, 111.08, 99.73, 79.37, 13.78. HRMS (ES) m/z calcd. for (C_17_H_15_N_3_O_2_; M + H)^+^ 294.1242 found 294.1240.

#### 3.2.5. *N*-Benzoyl-4-(pyrimidin-2-yl)piperazine-1-carboxamide (**5**)

*N*-(phenoxycarbonyl) benzamide (525 mg, 5.05 mmol) was condensed with 1-(2-Pyrimidyl) (315.8 mL, 2.22 mmol) in toluene (60 min reflux). The target compound was collected as solid, yield: (470 mg, 70%), m.p. 145–147 °C. ^1^H NMR (300 MHz, CDCl_3_) δ 8.75 (s, 1H, NH), 8.32 (d, *J* = 4.8 Hz, 2H, arom.CH), 7.63–7.51 (m, 1H, arom.CH), 7.47 (dd, *J* = 8.2, 6.7 Hz, 2H, arom.CH), 4.03–3.83 (m, 4H, CH_2_), 3.63 (t, *J* = 5.2 Hz, 4H, CH_2_). ^13^C NMR (75 MHz, CDCl_3_) δ 166.07, 161.65, 157.92, 153.78, 132.96, 132.85, 128.87, 128.03, 110.55, 43.57. HRMS (ES) m/z calcd. for (C_16_H_17_N_5_O_2_; M + H)^+^ 312.1460 found 312.1456.

#### 3.2.6. *N*-Benzoyl-4-(pyridin-4-yl)piperazine-1-carboxamide (**6**)

*N*-(phenoxycarbonyl) benzamide (590 mg, 2.44 mmol) and 1-(4-pyridyl)piperazine (411.51 mg, 2.51 mmol) were reacted in toluene (60 min reflux). This reaction afforded a brown solid, yield: (700 mg, 92%), m.p. 161–163 °C. ^1^H NMR (300 MHz, CDCl_3_) δ 9.05 (bs, 1H, NH), 8.33–8.25 (m, 2H, arom.CH), 7.95–7.86 (m, 2H, arom.CH), 7.64–7.53 (m, 1H, arom.CH), 7.48 (dd, *J* = 8.2, 6.8 Hz, 2H, arom.CH), 6.70–6.61 (m, 2H, arom.CH), 3.75–3.66 (m, 4H, CH_2_), 3.50–3.40 (m, 4H, CH_2_). ^13^C NMR (75 MHz, CDCl_3_) δ 166.22, 154.57, 153.75, 150.38, 133.00, 132.60, 129.49, 128.80, 127.96, 115.70, 108.58, 45.72. HRMS (ES) m/z calcd. for (C_17_H_18_N_4_O_2_; M + H)^+^ 311.1508 found 311.1505.

#### 3.2.7. *N*-Benzoyl-4-hydroxypiperazine-1-carboxamide (**7**)

*N*-(phenoxycarbonyl) benzamide (620 mg, 2.67 mmol) and 4-hydroxypiperidine (270 mg, 3.48 mmol) were stirred in toluene (60 min reflux). The pure product was a white solid, yield: (585 mg, 91%), m.p. 170–172 °C. ^1^H NMR (300 MHz, DMSO-d_6_) δ 10.11 (s, 1H, NH), 7.92–7.81 (m, 2H, arom.CH), 7.65–7.54 (m, 1H, arom.CH), 7.55–7.44 (m, 2H, arom.CH), 4.79 (d, *J* = 4.1 Hz, 1H, OH), 3.367–3.70 (m, 1H, CH), 3.21–3.12 (m, 2H, CH_2_), 2.52 (m, 2H, CH_2_), 1.75 (m, 2H,), 1.38 (m, 2H, CH_2_). ^13^C NMR (75 MHz, DMSO-d_6_) δ 166.60, 152.95, 133.78, 132.62, 128.86, 128.44, 65.80, 34.52. HRMS (ES) m/z calcd. for (C_13_H_16_N_2_O_3_; M + H)^+^ 249.1234 found 249.1235.

#### 3.2.8. *N*-Benzoyl-4-(p-tolyl)piperazine-1-carboxamide (**8**)

*N*-(phenoxycarbonyl) benzamide (510 mg, 2.11 mmol) dissolve (20 mL) and 1-(4-methylphenyl) piperazine (400 mg, 2.22 mmol) were coupled in toluene (60 min reflux). The filtered pure product was a crystalline white solid, yield: (490 mg, 72 %), m.p. 158–160 °C. ^1^H NMR (300 MHz, CDCl3) δ 8.53 (s, 1H, NH), 7.93–7.81 (m, 2H, arom.CH), 7.65–7.52 (m, 1H, arom.CH), 7.54–7.41 (m, 2H, arom.CH), 7.14–7.05 (m, 2H, arom.CH), 6.92–6.80 (m, 2H, arom.CH), 3.72 (s, 4H, CH_2_), 3.26–3.15 (m, 4H, CH_2_), 2.28 (s, 3H, CH_3_) ^13^C NMR (75 MHz, CDCl_3_) δ 166.01, 153.30, 149.00, 132.99, 132.92, 130.30, 129.90, 129.00, 128.94, 127.95, 117.27, 50.11, 20.60. HPLC/MS *t*_R_ 5.02 min.; m/z calcd. for (C_19_H_21_N_3_O_2;_ M+) 323.1634 found 323.9460.

#### 3.2.9. Tert-butyl 4-(3-benzoylureido)piperidine-1-carboxylate (**9**)

*N*-(phenoxycarbonyl) benzamide (740 mg, 3.07 mmol) and 4-amino-1-Boc-piperidine (510 mg, 3.48 mmol) were stirred in toluene (40 min reflux). The target product was obtained as a white solid, yield: (563 mg, 71%), m.p. 164–166 °C. ^1^H NMR (300 MHz, CDCl_3_) δ 8.95 (s, 1H, NH), 8.73 (d, *J* = 7.5 Hz, 1H, NH), 7.95–7.85 (m, 2H, arom.CH), 7.66–7.55 (m, 1H, arom.CH), 7.49 (dd, *J* = 8.3, 6.9 Hz, 2H, arom.CH), 4.13–3.83 (m, 2H, CH_2_), 2.96 (t, *J* = 12.3 Hz, 2H, CH_2_), 1.98 (dd, *J* = 13.0, 3.8 Hz, 2H, CH_2_), 1.47 (s, 11H, CH). ^13^C NMR (75 MHz, CDCl_3_) δ 168.00, 154.74, 153.19, 133.23, 132.33, 129.64, 128.91, 127.56, 115.33, 79.73, 47.38, 42.02, 31.83, 28.45. HRMS-ES) m/z calcd. for (C_18_H_25_N_3_O_4_; M + H)^+^ 348.1923 found 348.1880.

#### 3.2.10. *N*-(Benzo[d]oxazol-2-ylcarbamoyl)benzamide (**10**)

*N*-(phenoxycarbonyl) benzamide (571 mg, 2.36 mmol) and 2-amino benzoxazole (340 mg, 2.43 mmol) were condensed in at 25 °C in toluene (60 min reflux). The purified suspension afforded a white solid, yield: (551 mg, 83%), m.p. 180–182 °C. ^1^H NMR (300 MHz, DMSO-d_6_) δ 11.74 (s, 1H, NH), 8.03 (dd, *J* = 7.1, 1.8 Hz, 2H, arom.CH), 7.75–7.52 (m, 5H, arom.CH), 7.40–7.22 (m, 2H, arom.CH). ^13^C NMR (75 MHz, DMSO-d_6_) δ 167.59, 155.06, 148.41, 147.68, 140.00, 139.32, 133.37, 132.30, 128.82, 128.25, 124.83, 123.83, 118.11, 110.26. HRMS (ES) m/z calcd. for (C_15_H_11_N_3_O_3_; M + H)^+^ 282.0873 found 282.0866.

#### 3.2.11. *N*-(Benzoylcarbamoyl)-6-hydroxy-2,5,7,8-tetramethylchromane-2-carboxamide (**11**)

*N*-(phenoxycarbonyl) benzamide (510 mg, 2.11 mmol) was reacted with 6-hydroxy-2,5,7,8-tetramethylchromane-2-carboxamide (554 mg, 2.22 mmol) in toluene (60 min reflux). The reaction mixture yielded a white solid, yield: (605 mg, 72 %), m.p. 155–157 °C. ^1^H NMR (300 MHz, CDCl_3_) δ 8.50 (s, 1H, NH), 7.96–7.86 (m, 2H, arom.CH), 7.69–7.57 (m, 1H, arom.CH), 7.52 (dd, *J* = 8.2, 6.7 Hz, 2H, arom.CH), 6.36 (s, 1H, OH), 2.63 (q, *J* = 6.1 Hz, 2H, CH_2_), 2.37 (dt, *J* = 12.3, 5.9 Hz, 1H, CH_2_), 2.15 (d, *J* = 12.7 Hz, 3H, CH_3_), 2.06 (s, 3H, CH_3_), 1.89 (ddd, *J* = 13.9, 8.1, 6.4 Hz, 1H, CH_2_), 1.57 (s, 3H, CH_3_). ^13^C NMR (75 MHz, CDCl_3_) δ 176.96, 164.91, 149.64, 148.57, 141.00, 133.39, 132.89, 129.11, 127.91, 126.27, 122.73, 118.42, 78.88, 29.16, 24.55, 20.47, 13.18, 12.32, 12.15. HRMS (ES) m/z calcd. for (C_22_H_24_N_2_O_5_; M + H)^+^ 397.1763 found 397.1759.

#### 3.2.12. *N*-((4-Hydroxy-3-methoxyphenylcarbonothioyl)carbamoyl) benzamide (**12**)

*N*-(phenoxycarbonyl) benzamide (478 mg, 1.98 mmol) and 4-hydroxy-3- methoxythiobenzamide (381 mg, 2.10 mmol) were reacted in toluene (30 min reflux). The pure product was collected as a yellow solid, yield: (420 mg, 64%), m.p. 176–177 °C. ^1^H NMR (300 MHz, DMSO-d_6_) δ 12.90–12.83 (m, 1H, arom.CH), 11.55 (s, 1H, arom.CH), 10.15 (s, 1H, OH), 8.02 (d, *J* = 8.0 Hz, 2H, arom.CH), 7.80–7.33 (m, 5H, arom.CH), 6.87 (d, *J* = 8.4 Hz, 1H, arom.CH), 3.85 (s, 3H, OCH_3_). ^13^C NMR (75 MHz, DMSO-d_6_) δ 169.23, 152.00, 147.64, 133.94, 133.68, 132.52, 129.21, 128.79, 121.90, 115.29, 112.68, 79.36, 56.13. HRMS (ES) m/z calcd. for (C_16_H_14_N_2_O_4_S; M + H)^+^ 331.0752 found 331.0745.

#### 3.2.13. *N*-((4,6-Dimethylpyrimidin-2-yl)carbamoyl)benzamide (**13**)

*N*-(phenoxycarbonyl) benzamide (1.22 g, 5.05 mmol) and 2-amino-4,6-dimethylpyrimidine (650 mg, 5.27 mmol) were condensed in toluene (30 min reflux). The suspension obtained after being cooled down the reaction mixture was collected by filtration and dried. The pure product was collected by filtration and dried under a vacuum. White crystalline solid, yield: (913 mg, 68%), m.p. 183–185 °C. ^1^H NMR (300 MHz, CDCl_3_) δ 8.09–7.98 (m, 2H, arom.CH), 7.67–7.55 (m, 1H, arom.CH), 7.58 –7.45 (m, 2H, arom.CH), 6.77 (s, 1H, arom.CH), 2.48 (s, 6H, 2CH_3_). ^13^C NMR (75 MHz, CDCl_3_) δ 156.87, 149.58, 133.00, 128.79, 127.89, 23.85. HRMS (ES) m/z calcd. for (C_14_H_14_N_4_O_2_; M + H)^+^ 271.1195 found 271.1188.

#### 3.2.14. *N*-(Quinolin-3-ylcarbamoyl)benzamide (**14**)

*N*-(phenoxycarbonyl) benzamide (460 mg, 1.90 mmol) dissolved in toluene (20 mL) and 3-aminoquinoline (291 mg, 2.0 mmol) were coupled in toluene (60 min reflux). The resulting pure product was a white fluffy solid, yield: (505 mg, 94 %), m.p. 233–236 °C. ^1^H NMR (300 MHz, DMSO-d_6_) δ 11.27 (s, 1H, NH), 11.15 (s, 1H, NH), 9.00 (d, *J* = 2.5 Hz, 1H, arom.CH), 8.69 (d, *J* = 2.6 Hz, 1H, arom.CH), 8.14–7.91 (m, 4H, arom.CH), 7.75–7.49 (m, 5H, arom.CH). ^13^C NMR (75 MHz, DMSO-d_6_) δ 169.19, 152.13, 145.68, 144.93, 133.65, 132.68, 132.03, 129.10, 129.07, 128.84, 128.58, 128.24, 128.22, 127.65, 123.62. HRMS (ES) m/z calcd. for (C_17_H_13_N_3_O_2_; M + H)^+^ 292.1086 found 292.1082.

#### 3.2.15. *N*-(Isoquinolin-3-ylcarbamoyl)benzamide (**15**)

*N*-(phenoxycarbonyl) benzamide (608 mg, 2.52 mmol) and 3-aminoisoquinoline (430 mg, 2.98 mmol) were coupled in toluene (30 min reflux). The purified desired compound was a white solid, yield: (691 mg, 94 %), m.p. 237–238 °C. ^1^H NMR (300 MHz, DMSO-d_6_) δ 11.45 (s, 1H, NH), 11.28 (s, 1H, NH), 9.18 (d, *J* = 1.1 Hz, 1H, arom.CH), 8.42 (s, 1H, arom.CH), 8.14–8.01 (m, 3H, arom.CH), 7.98 (dd, *J* = 8.5, 1.1 Hz, 1H, arom.CH), 7.81–7.62 (m, 2H, arom.CH), 7.62–7.51 (m, 3H, arom.CH). ^13^C NMR (75 MHz, DMSO-d_6_) δ 169.39, 152.26, 151.31, 146.56, 137.69, 133.61, 132.77, 131.61, 129.05, 128.88, 128.19, 126.89, 126.46, 126.38, 107.06. HRMS (ES) m/z calcd. for (C_17_H_13_N_3_O_2;_ M + H)^+^ 292.1086 found 292.1083.

#### 3.2.16. *N*-(Isoquinolin-3-ylcarbamothioyl)benzamide (**16**)

Benzoyl isothiocyanate (1.0 mL, 8.37 mmol) was added dropwise to 3-aminoisoquinoline (1.2 gm, 8.33 mmol) dissolved in THF (20 mL) at room temperature. The reaction mixture was refluxed for 2 h, cooled, and filtered. The yellow solid obtained was further purified by flash chromatography DCM/MeOH (9:1) to afford a pale yellow solid, yield: (1.5 gm, 65%), m.p. 162–165 °C. ^1^H NMR (300 MHz, CDCl_3_) δ 13.35 (s, 1H, NH), 9.29 (s, 1H, NH), 9.10 (s, 2H, arom.CH), 8.00–7.85 (m, 4H, arom.CH), 7.75–7.48 (m, 6H, arom.CH). ^13^C NMR (75 MHz, CDCl_3_) δ 176.24, 166.54, 151.55, 146.10, 137.23, 133.83, 131.82, 131.10, 129.32, 127.68, 127.65, 127.40, 127.03, 110.89. HPLC/MS *t*_R_ 5.76 min.; m/z calcd for (C_17_H_13_N_3_OS_;_ M+) 307.0779 found 307.8182.

### 3.3. Molecular Docking

The docking protocol included: deriving two-dimension (2D) structures of target compounds by ChemDraw Ultra 19.1 and saving them as structural data files (SDFs) prior to converting them into three-dimension (3D) images with openBabel 3.1.1 program [35] in PyRx [15]. Energy minimized conformers were then converted into Protein Data Bank, Partial Charge (Q), and Atom Type (T) or *pdbqt* format. The σ1R/PD-14418 complex structure was obtained from the Protein Data Bank database (http://www.rcsb.org/pdb, PDB, ID: 5HK1), and imported into Discovery Studio software for visualizing the binding domain of the complex and identification of the amino acid (AA) residues in the binding pocket. Hydrogen atoms were added to the protein to correct the ionization and tautomeric states of amino acid residues. Furthermore, H_2_O molecules and complexes bound to σ1R were removed before the docking. In addition, the protein was subjected to energy minimization by applying the UFF force field, with a maximum number of 200 steps at an RMS gradient of 0.02. Amino acids within 8Å of the binding sites were used for docking studies in Autodock vina [16]. The optimized protein was saved in *pdbqt* format and imported to the PyRx workstation for molecular docking via Autodock Vina virtual screening tool.

The auto grid generated by vina wizard in PyRx was utilized for arrangement affiliations of grid maps and contained 1.00 Å spacing with box dimensions of 11.78X × 37.22Y × −34.86Z Å and centers of 76.160X × 71.016Y × 71.999Z Å. The docking grid box was centered to cover residues within the active site in the protein structure. The validation of the docking study was performed by re-docking PD14418 into an appropriate protein cavity. Re-docking was accepted if the root mean square deviation (RMSD) was <2.0 Å. Hydrophobic and hydrogen bond interactions between the docked compounds and protein were investigated utilizing the discovery studio software.

### 3.4. In Vitro σ1R Binding Assay

Sigma-1 binding affinity experiments (K_i_ determinations) were conducted at the National Institute of Mental Health’s Psychoactive Drug Screening Program (NIMH PDSP). Sigma-1 receptor binding assay protocol details (radioligand displacement conditions, etc.) are available at https://pdsp.unc.edu/pdspweb/content/PDSP%20Protocols%20II%202013-03-28.pdf (accessed 28 February 2023) [30]. The K_i_ binding affinity values of our target compounds are also displayed in Table 4.

## 4. Conclusions

Sixteen acyl ureas (i.e., benzoyl ureas and benzoyl thioureas) were rationally designed, and readily synthesized in good to high yields (64–94%). Per the SciFinder database, fourteen of these compounds are new. This compound series exhibited drug-like properties (complied with Ro5 and TPSA/NRB parameters), possessed modeled σ1R affinities (−8.6 to −11.4 kcal/mol) in the range comparable to the reference PD14418 (−10.1 kcal/mol), and the in vitro σ1R binding data revealed two compounds (**10** and **12,** with respective K_i_ values of 2.18 and 9.54 μM) as promising leads. The two new leads have now opened the door for optimization in our follow-up structure-activity relationship (SAR) studies in search of potent σ1R acyl urea ligands with anti-neurodegenerative applications.

## Figures and Tables

**Table 1:** Synthesized acyl ureas plus starting amines/amide/thioamides. *^a^* Characterized by ^1^H, ^13^C NMR, HRMS. *^b^* Yields of pure isolated products. ** Known compounds.

Compds.	Amine	Acyl Urea *^a^*	% Yield *^b^*	MP (°C)
**1**	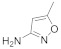	** [25,26] 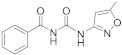	76	212–215 (Not reported)
**2**	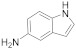	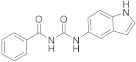	94	220–223
**3**	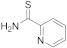	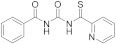	75	180–182
**4**	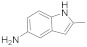	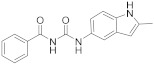	71	209–211
**5**	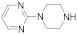	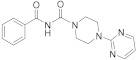	70	145–147
**6**	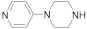	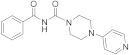	92	161–163
**7**	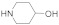	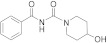	91	170–172
**8**	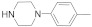	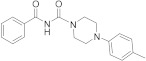	72	158–160
**9**	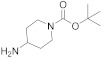	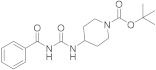	71	164–166
**10**	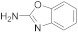	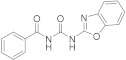	83	180–182
**11**	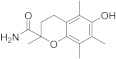	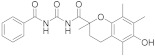	72	155–157
**12**	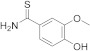	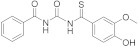	64	176–177
**13**	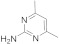	** [27] 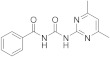	68	183–185 (Reported 184–186)
**14**	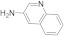	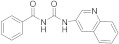	94	233–236
**15**	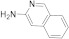	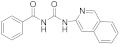	94	237–238
**16**	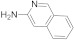	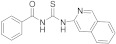	65	162–165

**Table 2:** Some Molinspiration/OSIRIS predicted pharmacokinetic properties. Calculated partition coefficients (cLogP values) were Octanol/H_2_O based.

Entry	MW	cLogP	TPSA	ON	OHNH	NRB	Drug-Likeness
1	231.2	1.75	84.23	6	2	2	1.02
2	279.3	2.63	73.99	5	3	2	−0.05
3	285.3	2.14	103.1	5	2	3	1.75
4	293.3	3.03	73.99	5	3	2	0.59
5	311.3	1.53	78.43	7	1	2	8.16
6	310.3	1.72	65.54	6	1	2	6.83
7	249.2	0.32	72.89	6	2	1	5.88
8	323.4	3.07	52.65	5	1	2	0.81
9	332.4	2.64	78.95	6	1	4	−27.5
10	281.2	2.97	84.23	6	2	2	2.54
11	396.4	3.60	87.66	7	3	2	1.80
12	330.3	2.67	119.7	6	3	4	2.42
13	270.2	2.19	83.98	6	2	2	1.62
14	291.3	2.91	71.09	5	2	2	1.66
15	291.3	3.14	71.09	5	2	2	1.66
16 Ro5	307.3 ≤500	3.53 ≤5	86.11	4 ≤10	2 ≤5	4	0.73

**Table 3:** PyRx generated binding affinity values, and the number of H-bonds and residue(s) involved in H-bonding.

Entries	Binding Affinity	#H-Bond Interactions	Residues Involved in 5HK1 H-Bond Interactions
1	−10.0	2	TYR 120, THR 181
2	−9.6	1	GLU 172
3	−9.6	1	GLU 172
4	−10.4	1	GLU 172
5	−10.0	1	TYR 120
6	−9.6	1	GLU 172
7	−8.6	1	GLU 172
8	−9.7	1	GLU 172
9	−9.0	1	GLU 172
10	−10.6	3	THR 181, TYR 108, GLU 172
11	−9.5	3	TYR 120, THR 181, ASP 126
12	−9.9	2	ASP 126, GLU 172
13	−10.3	3	GLU 213, SER 99, SER 33
14	−10.6	1	GLU 172
15	−11.4	0	-
16	−10.5	2	GLU 172, TYR 103
PD-14418	−10.2	0	-

**Table 4:** Modeled (kcal/mol) versus in vitro (μM) binding affinity values. * PD144418 is a selective and high affinity (K_i_ = 0.08 nM) σ1R ligand [33].

Entries	Calculated σ1R Affinity (kcal/mol)	In Vitro σ1R Affinity (Ki, μM)
1	−10.0	>10
2	−9.6	>10
3	−9.6	>10
4	−10.4	>10
5	−10.0	>10
6	−9.6	>10
7	−8.6	>10
8	−9.7	>10
9	−9.0	>10
10	−10.6	2.18
11	−9.5	>10
12	−9.9	9.54
13	−10.3	>10
14	−10.6	>10
15	−11.4	>10
16	−10.5	>10
PD-14418	−10.2	*

## Data Availability

Not applicable.

## Supplementary Materials

Compound ^1^HNMR and ^13^CNMR spectra are available online at https://www.mdpi.com/article/10.3390/molecules28052319/s1.
