# Supplementary material for: Design and Synthesis of New Acyl Urea Analogs as Potential σ1R Ligands
Source: Molecules. 2023 Mar 2;28(5):2319. doi: 10.3390/molecules28052319 (PMC10005056; doi:10.3390/molecules28052319)

^{1}H and ^{13}C NMR spectra of compounds 1 – 16.

^{1}H NMR of compound **1** (300 MHz, $CDCl_3$)

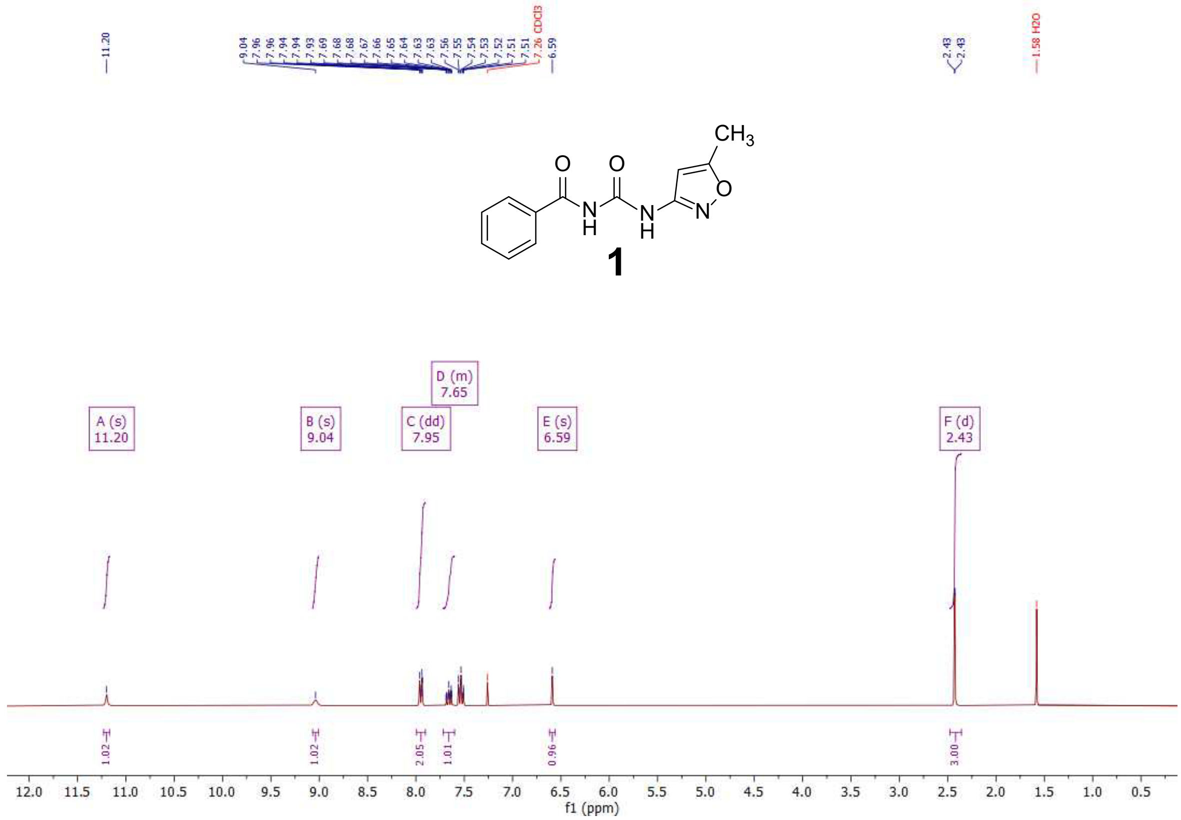

^{13}C NMR of compound **1** (75 MHz, $CDCl_3$)

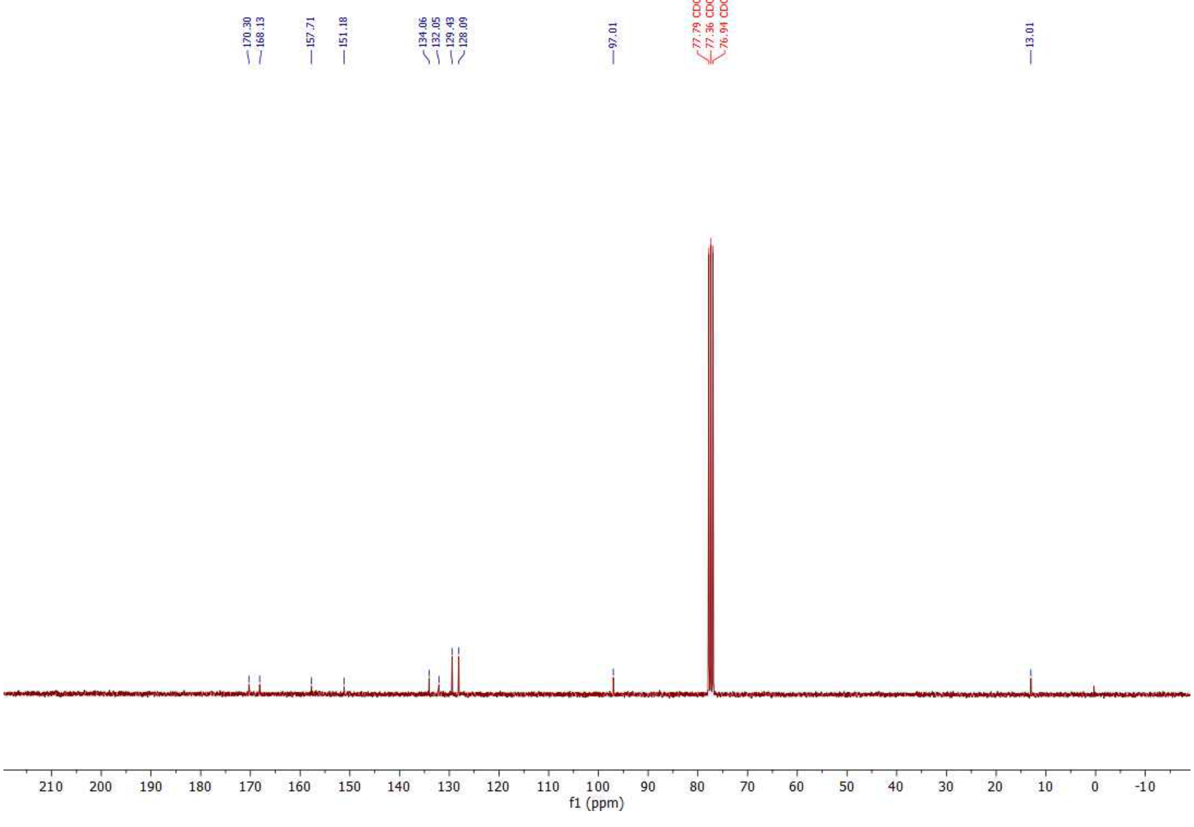

^{1}H NMR of compound **2** (300 MHz, dmso-d_6)

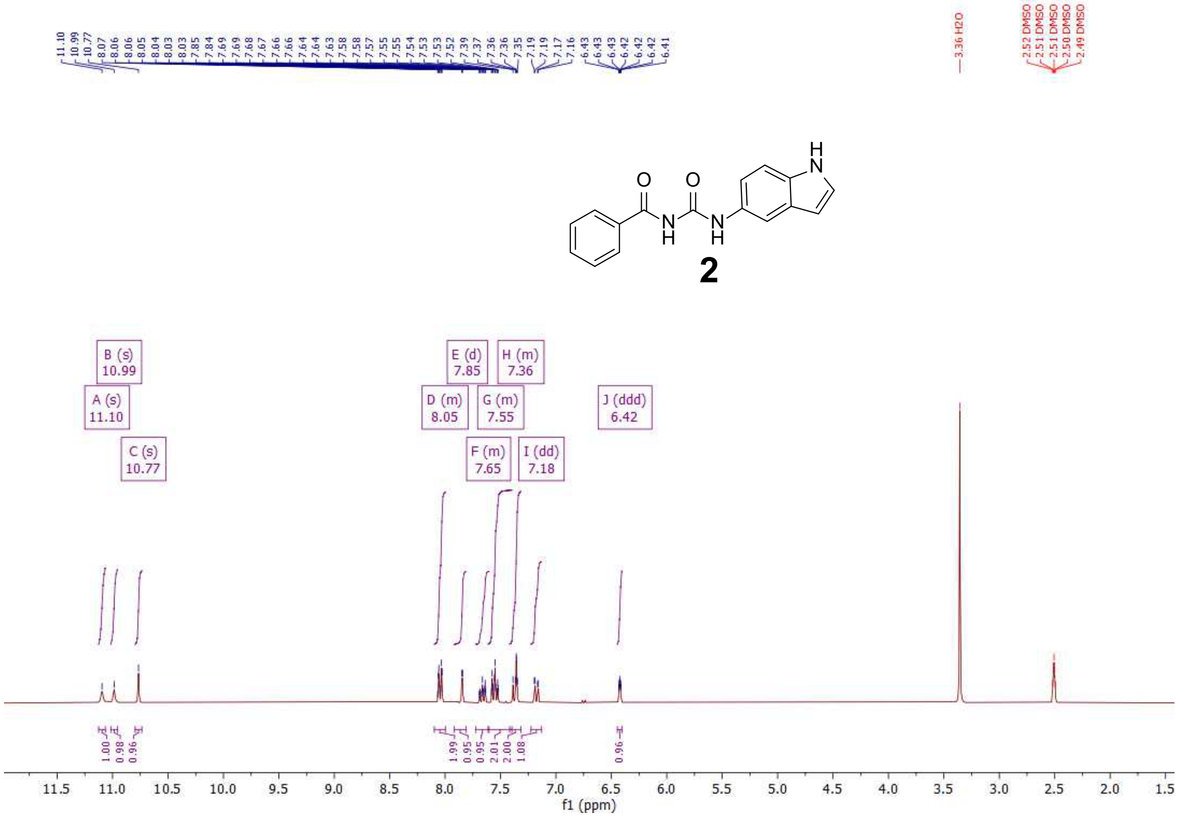

^{13}C NMR of compound **2** (75 MHz, dmso-d_6)

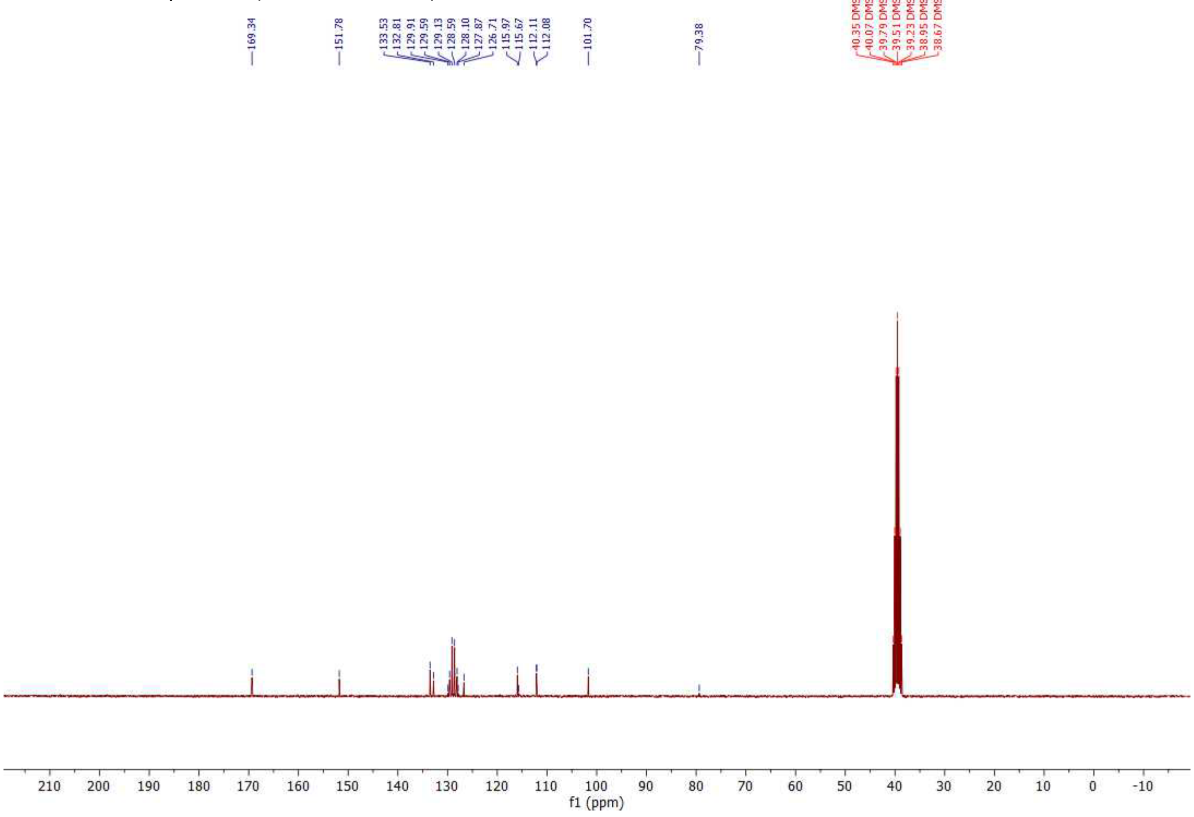

^{1}H NMR of compound **3** (300 MHz, dmso-d_6)

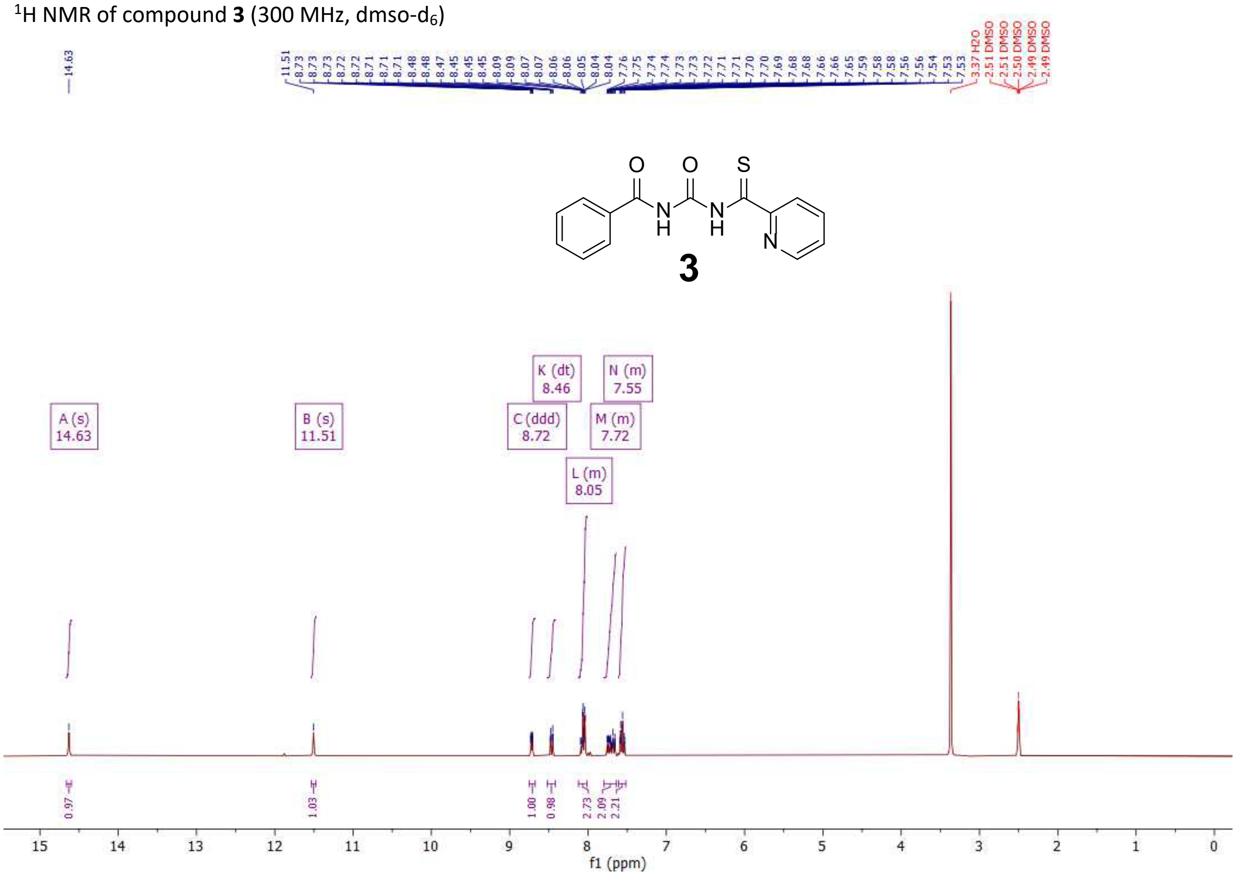

^{13}C NMR of compound 3 (75 MHz, dmso-d_6)

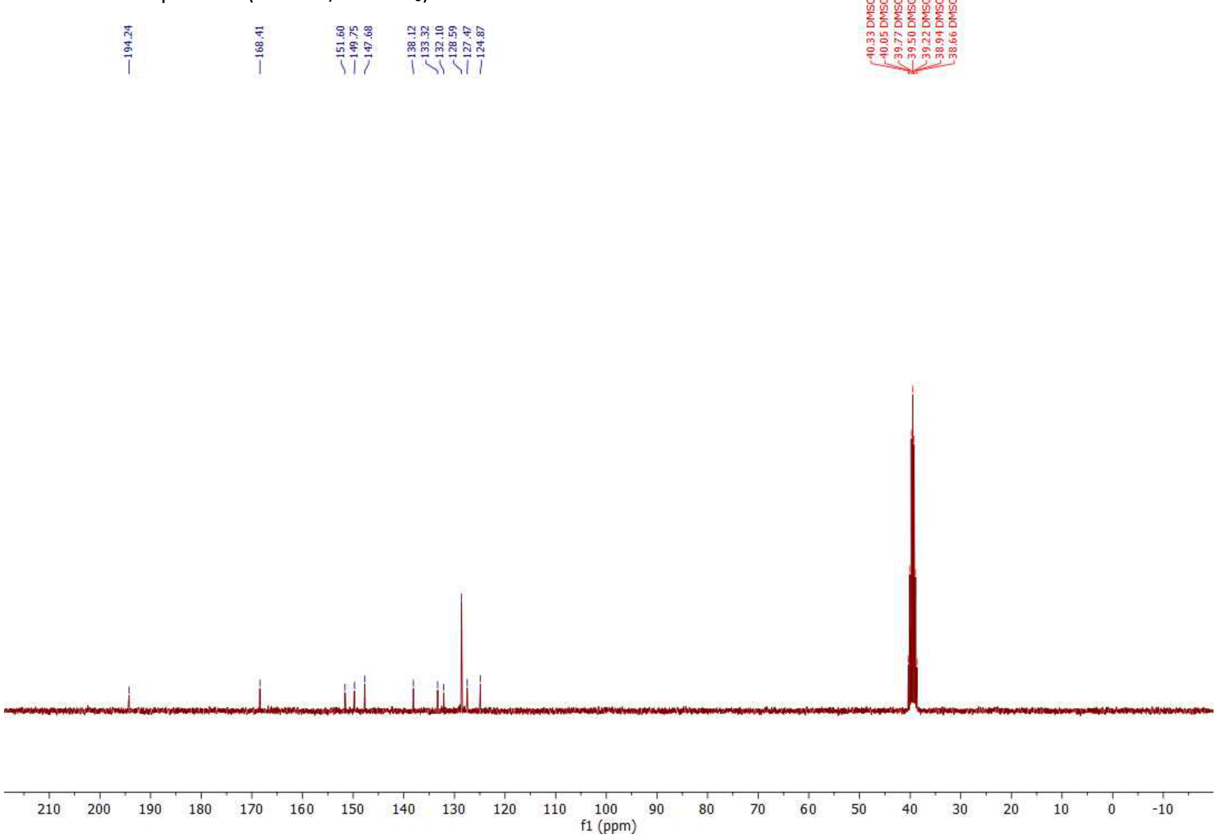

^{1}H NMR of compound **4** (300 MHz, dmso-d_6)

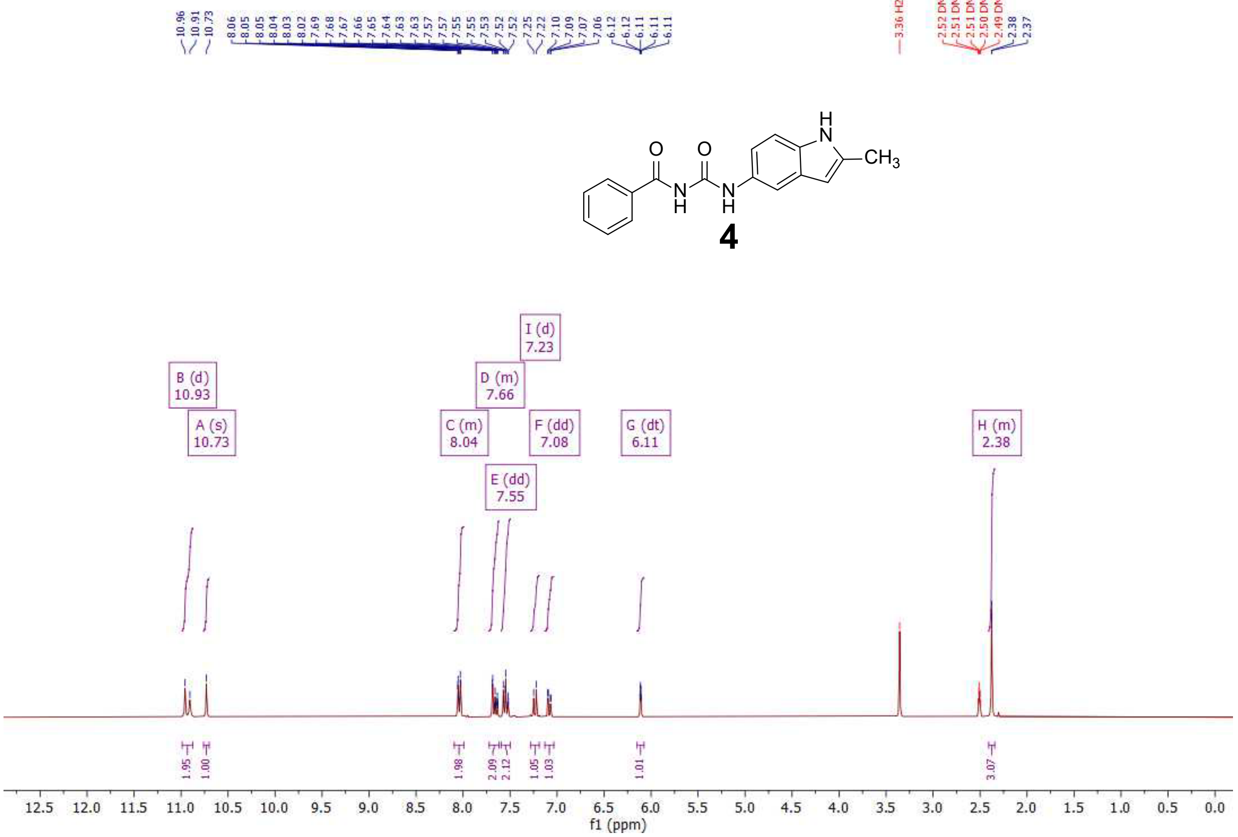

4

^{13}C NMR of compound **4** (75 MHz, dmso-d_6)

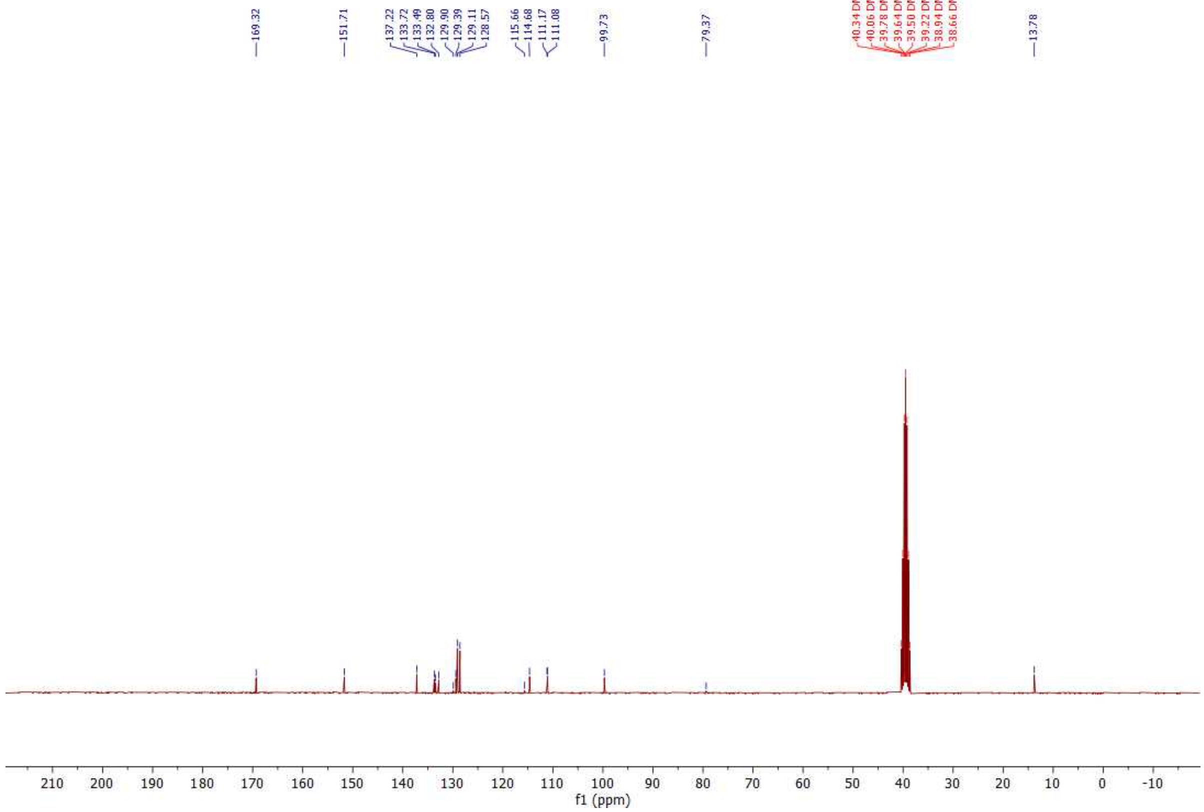

^{1}H NMR of compound **5** (300 MHz, $CDCl_3$)

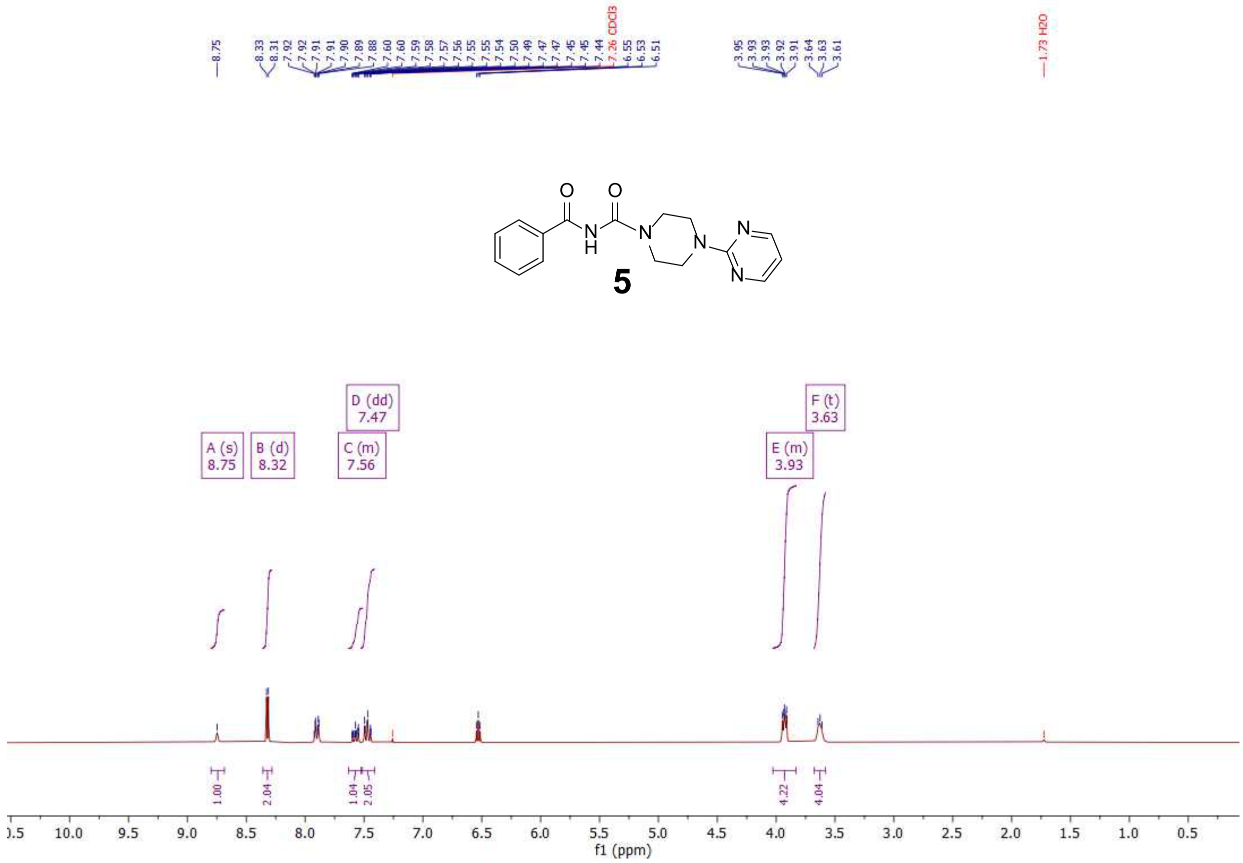

^{13}C NMR of compound **5** (75 MHz, $CDCl_3$)

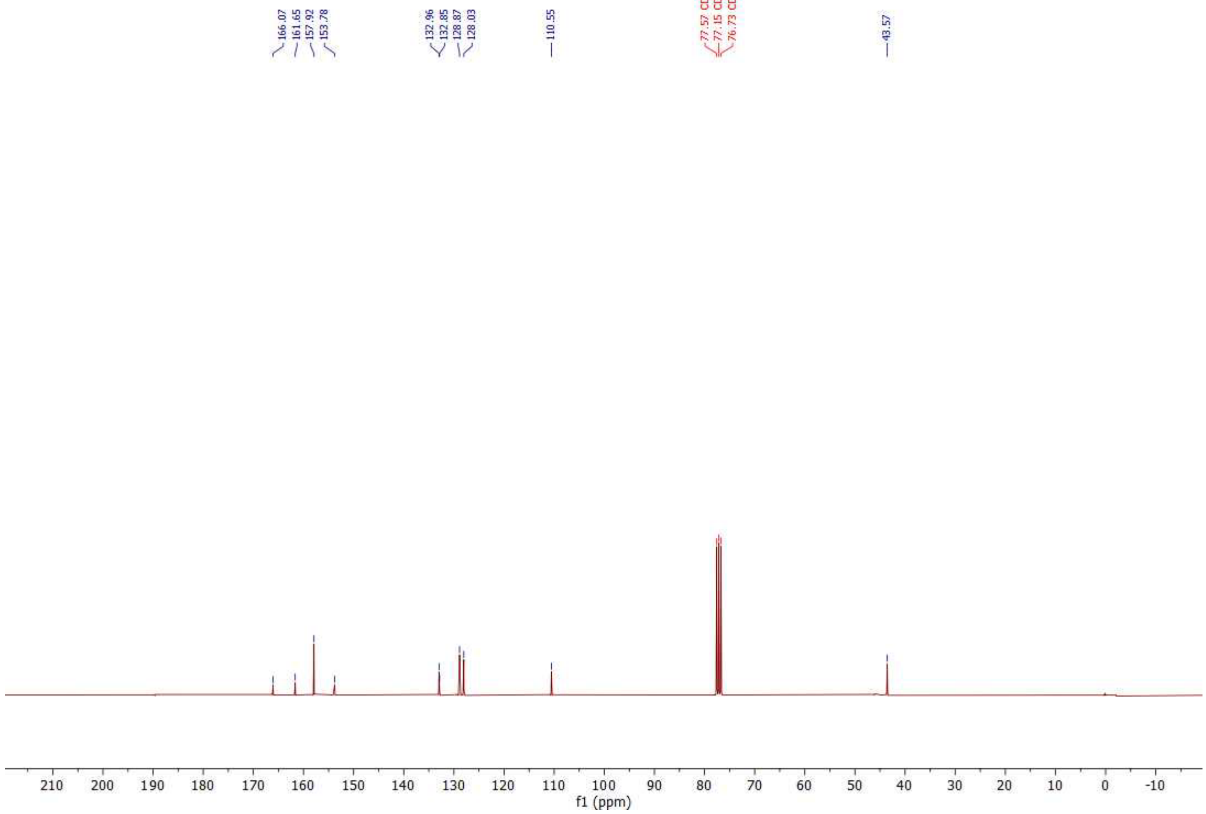

^{1}H NMR of compound **6** (300 MHz, $CDCl_3$)

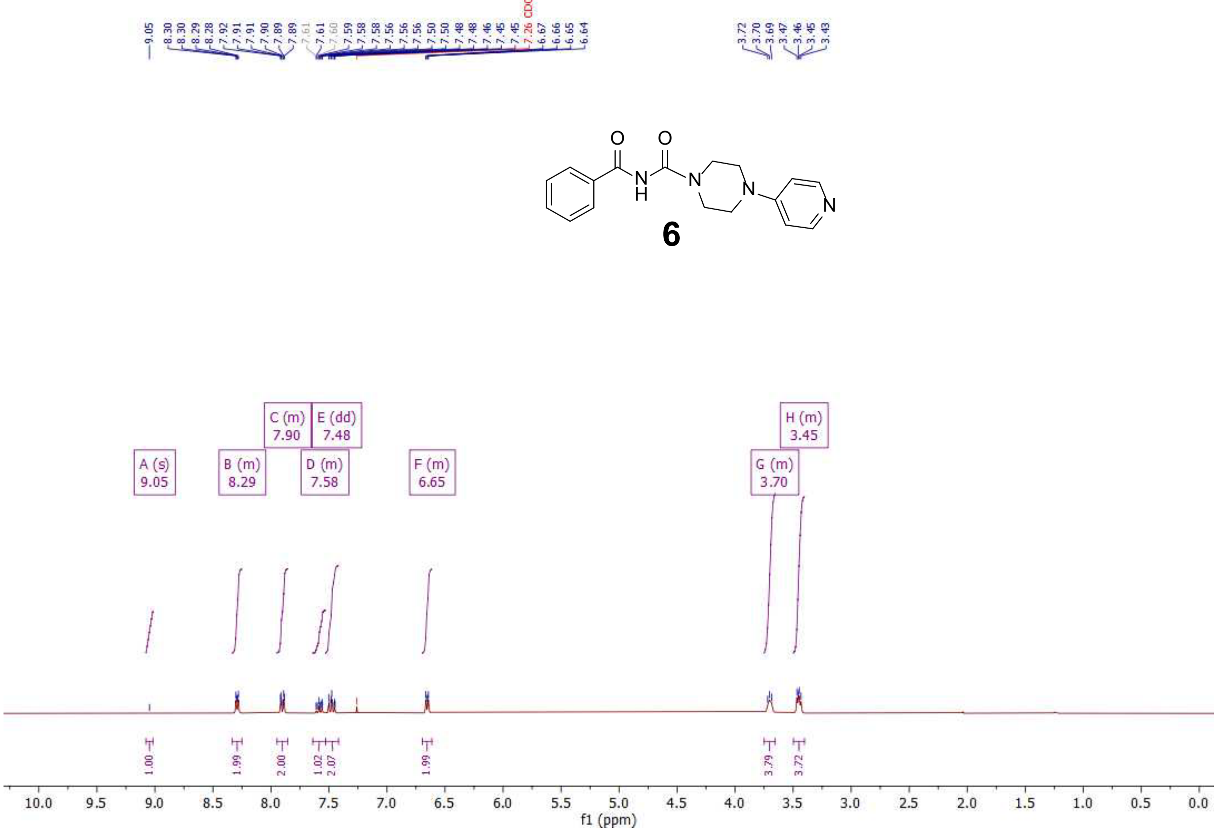

^{13}C NMR of compound **6** (75 MHz, $CDCl_3$)

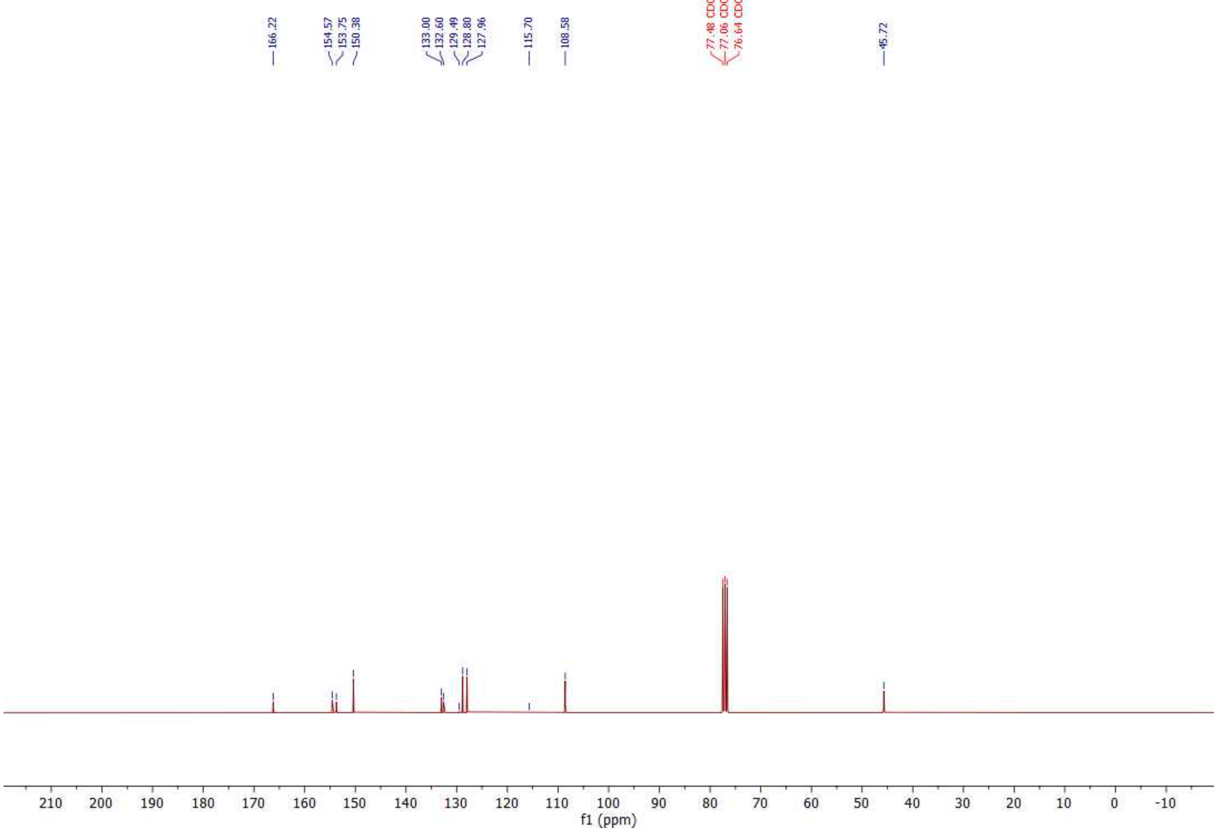

^{1}H NMR of compound **7** (75 MHz, dmso-d_6)

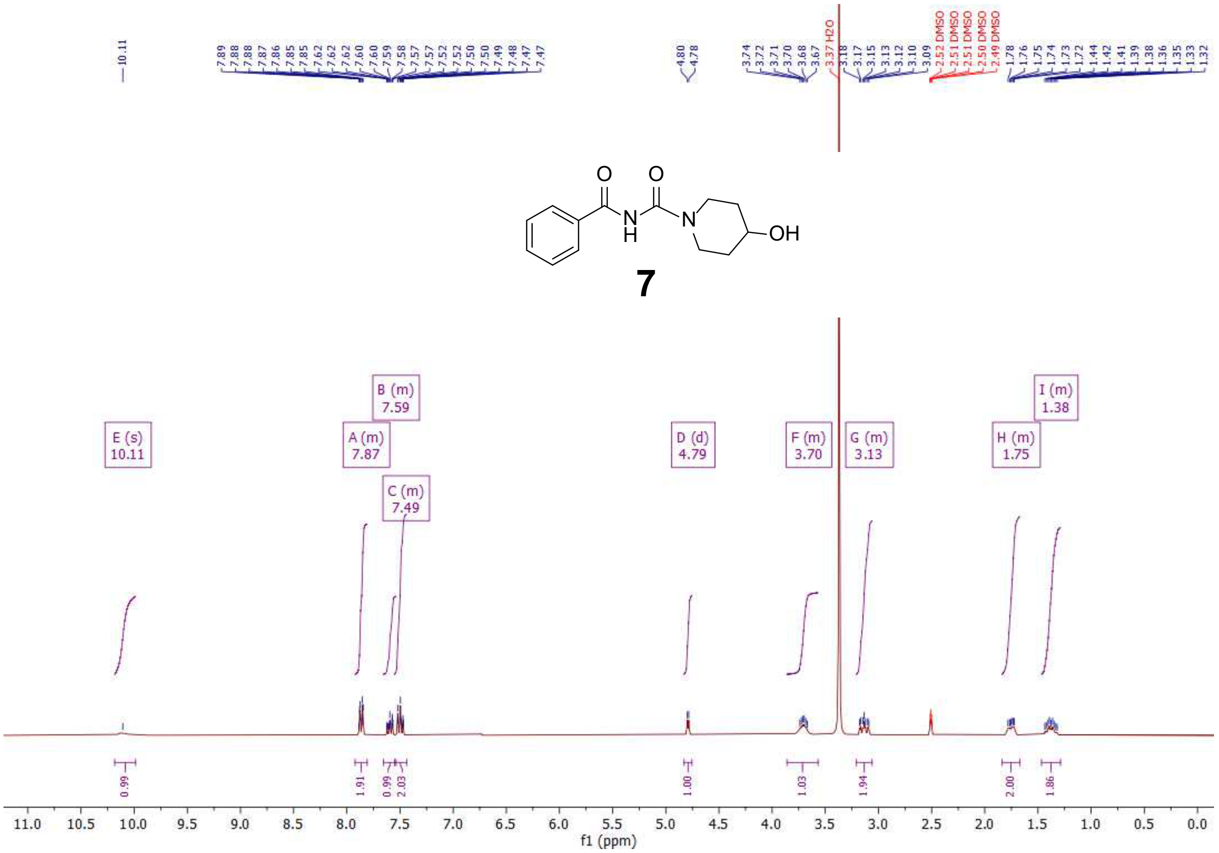

^{13}C NMR of compound **7** (75 MHz, dmso-d_6)

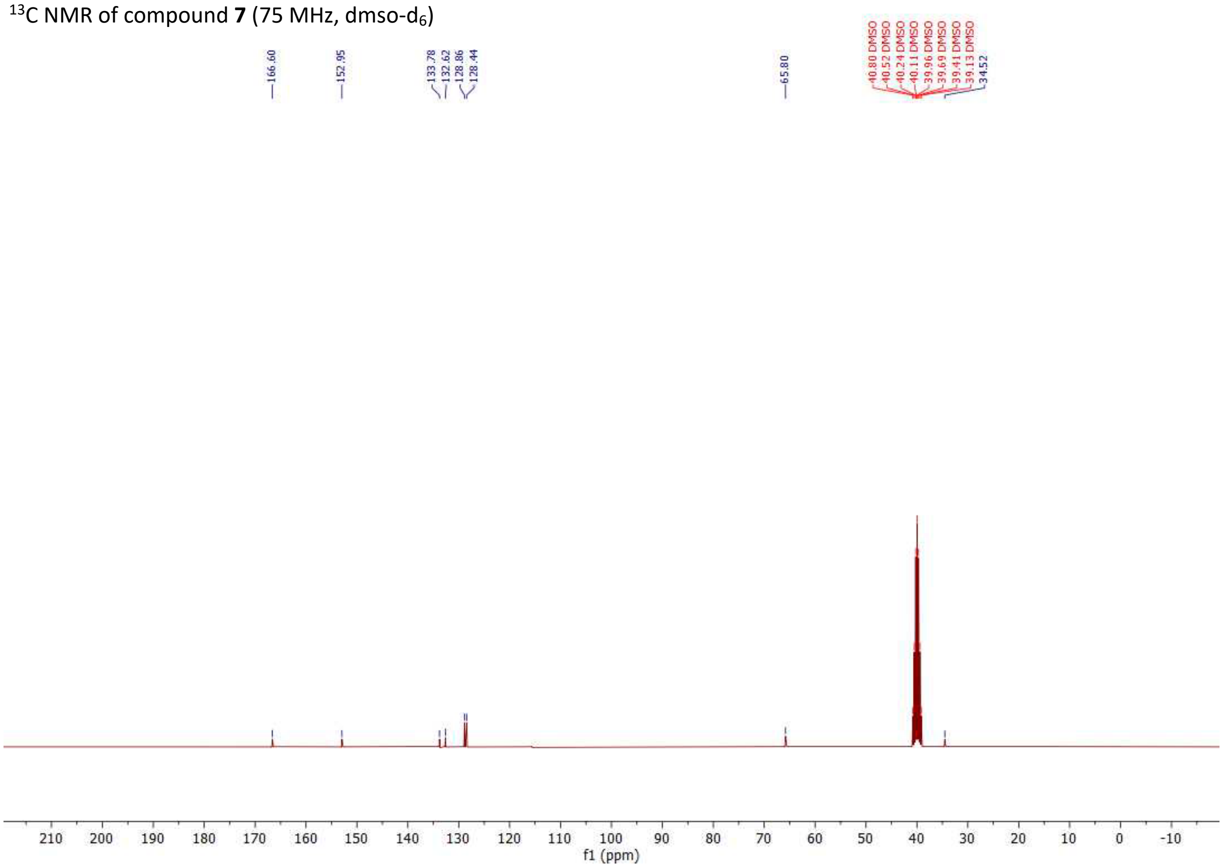

^{1}H NMR of compound **8** (300 MHz, $CDCl_3$)

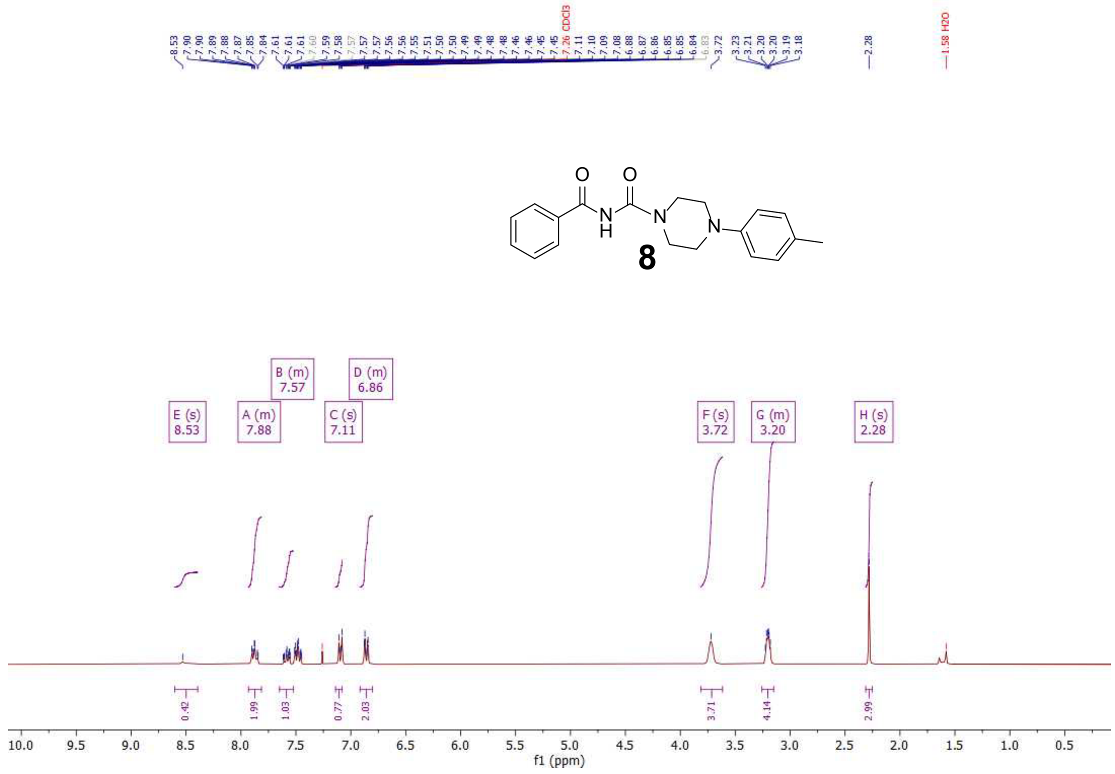

^{13}C NMR of compound **8** (75 MHz, $CDCl_3$)

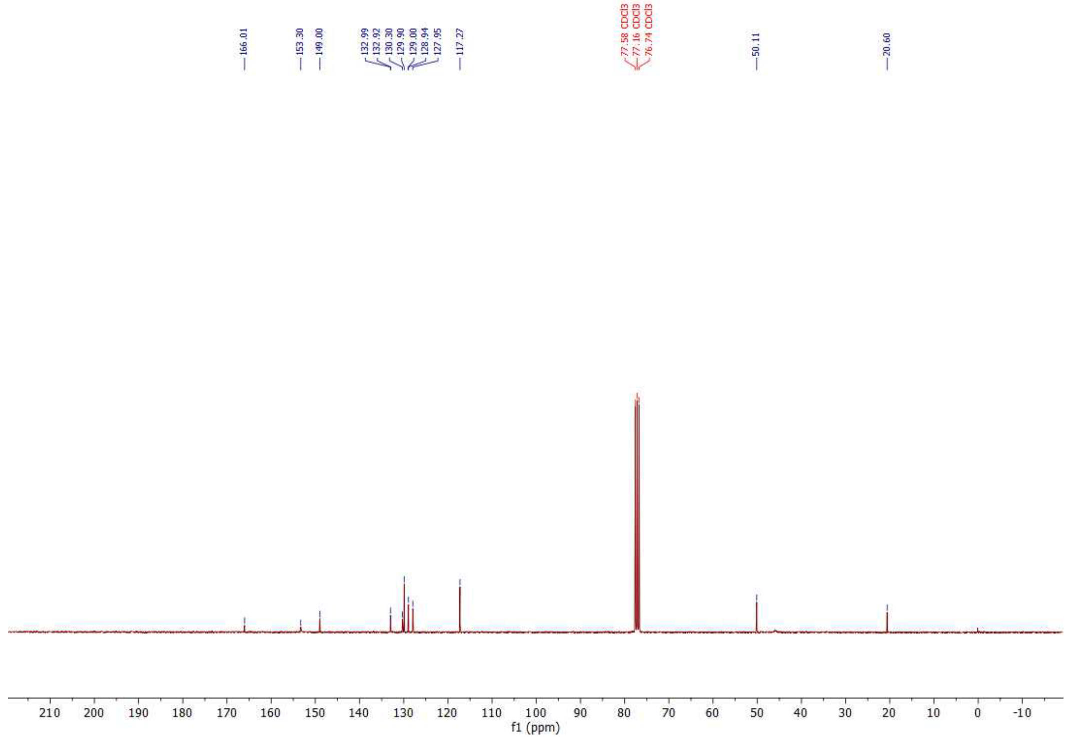

^{1}H NMR of compound **9** (300 MHz, $CDCl_3$)

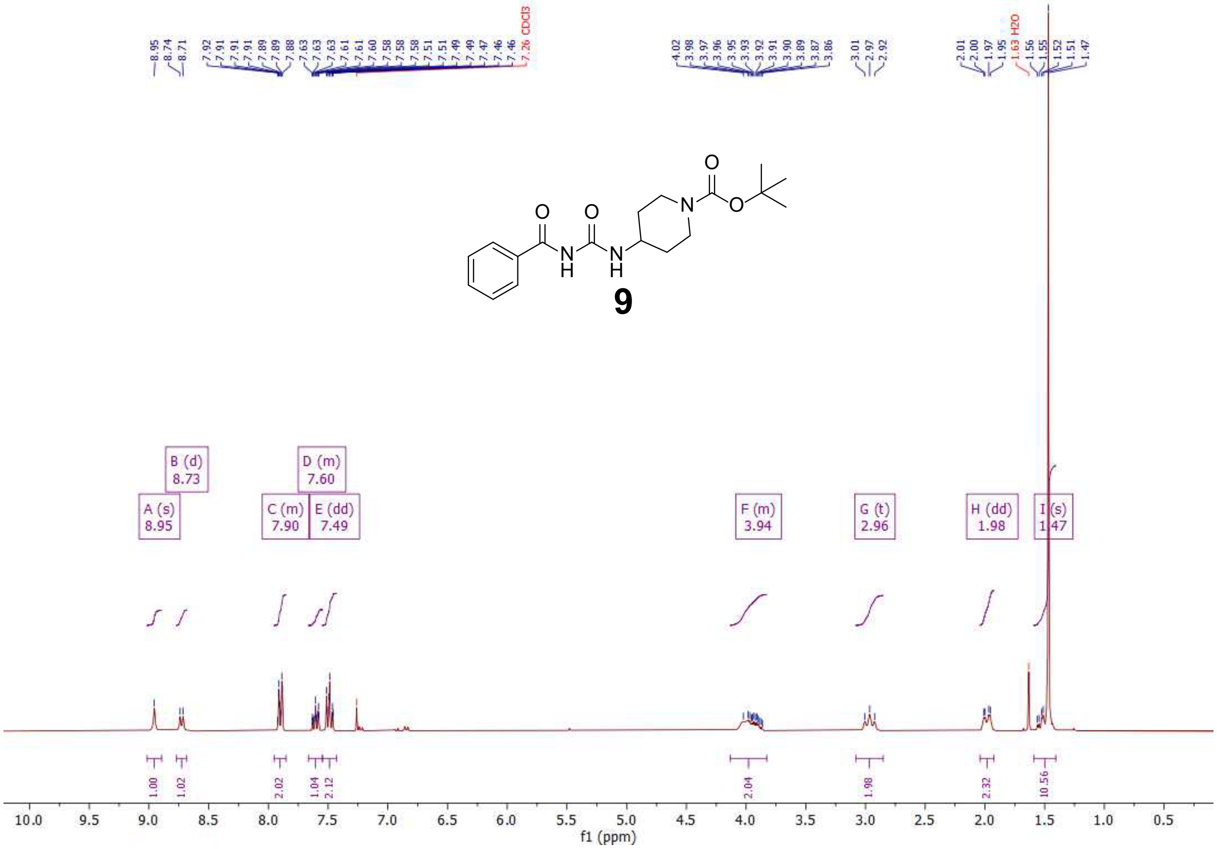

^{13}C NMR of compound **9** (75 MHz, $CDCl_3$)

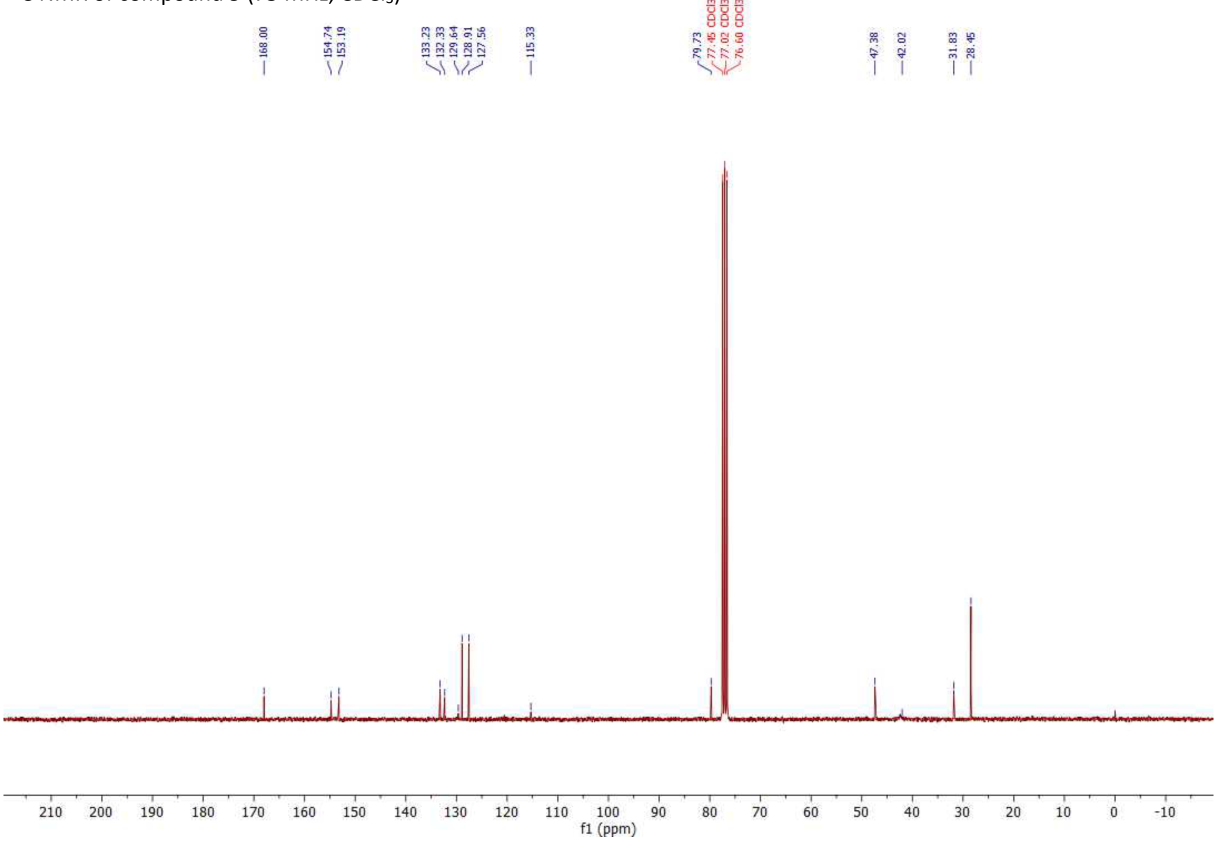

^{1}H NMR of compound **10** (300 MHz, dmso-d_6)

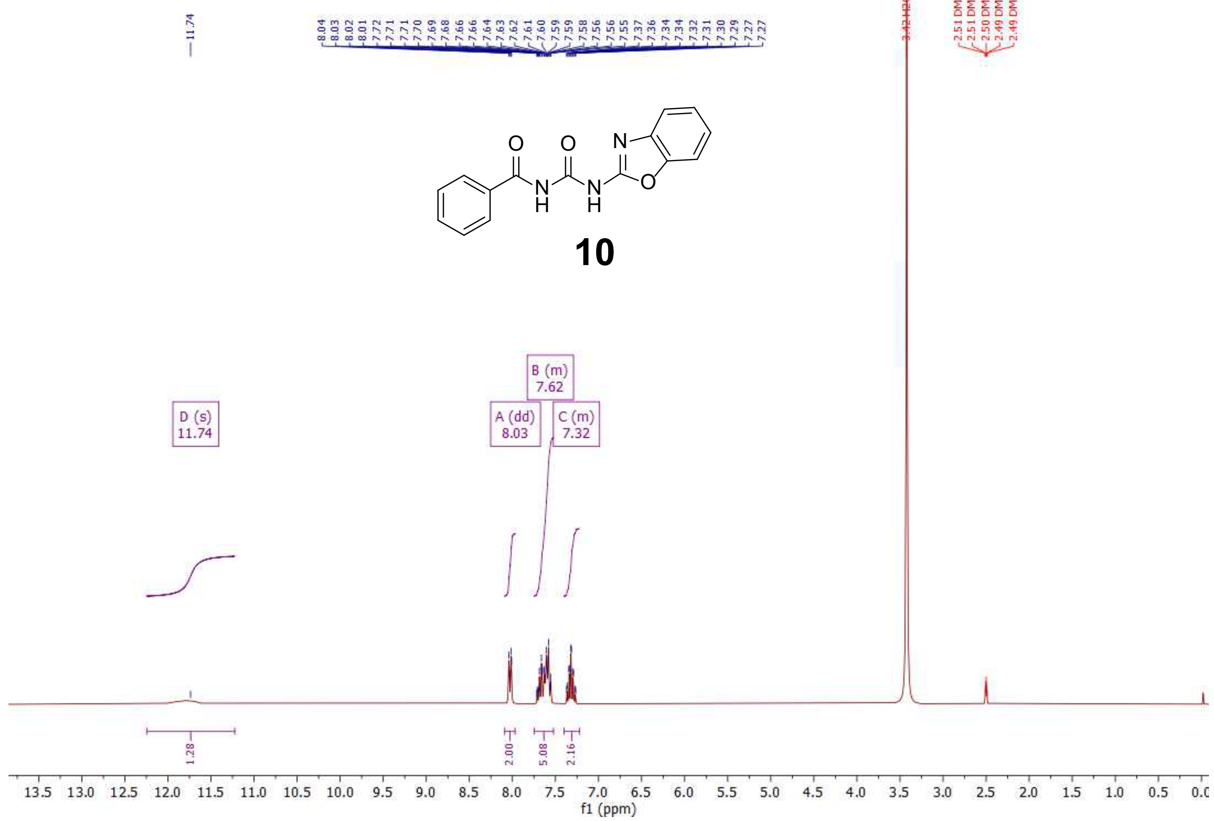

^{13}C NMR of compound **10** (75 MHz, dmso-d_6)

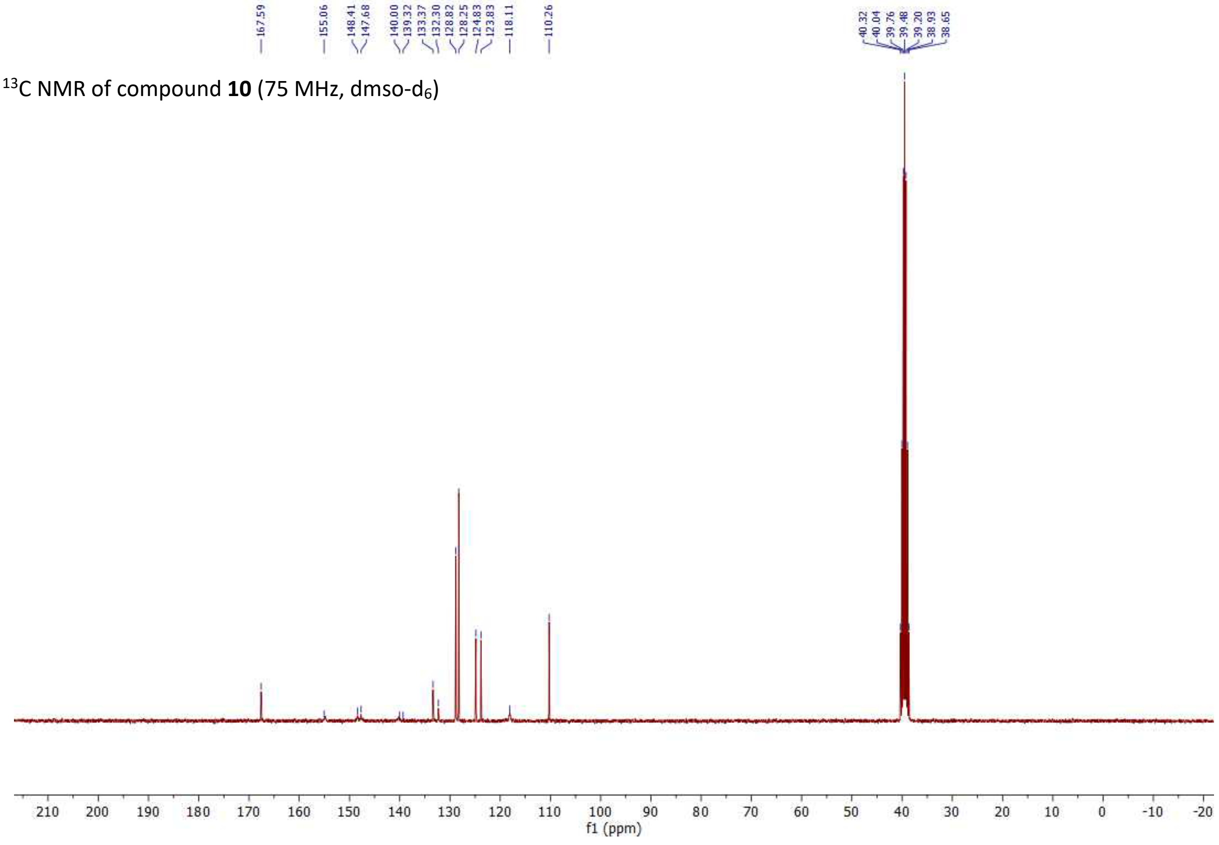

^{1}H NMR of compound **11** (300 MHz, $CDCl_3$)

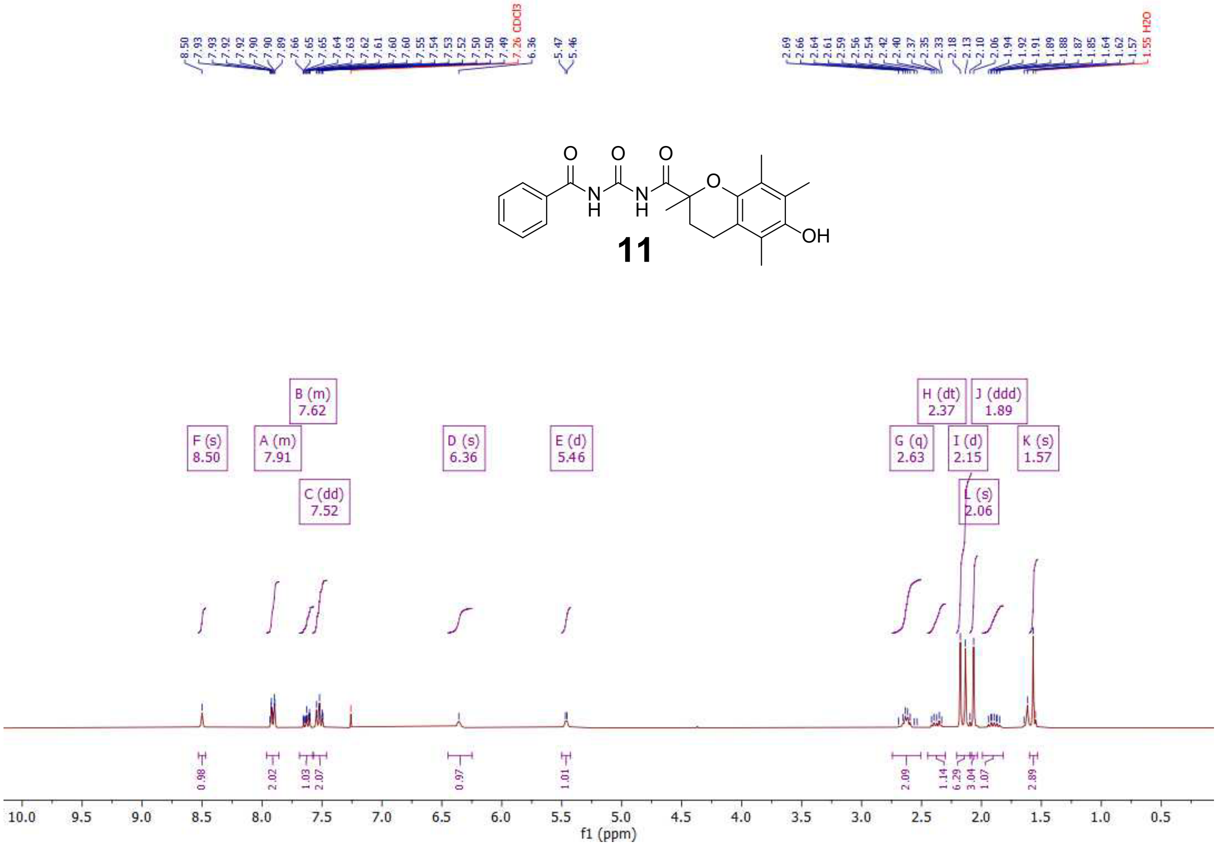

^{13}C NMR of compound **11** (75 MHz, $CDCl_3$)

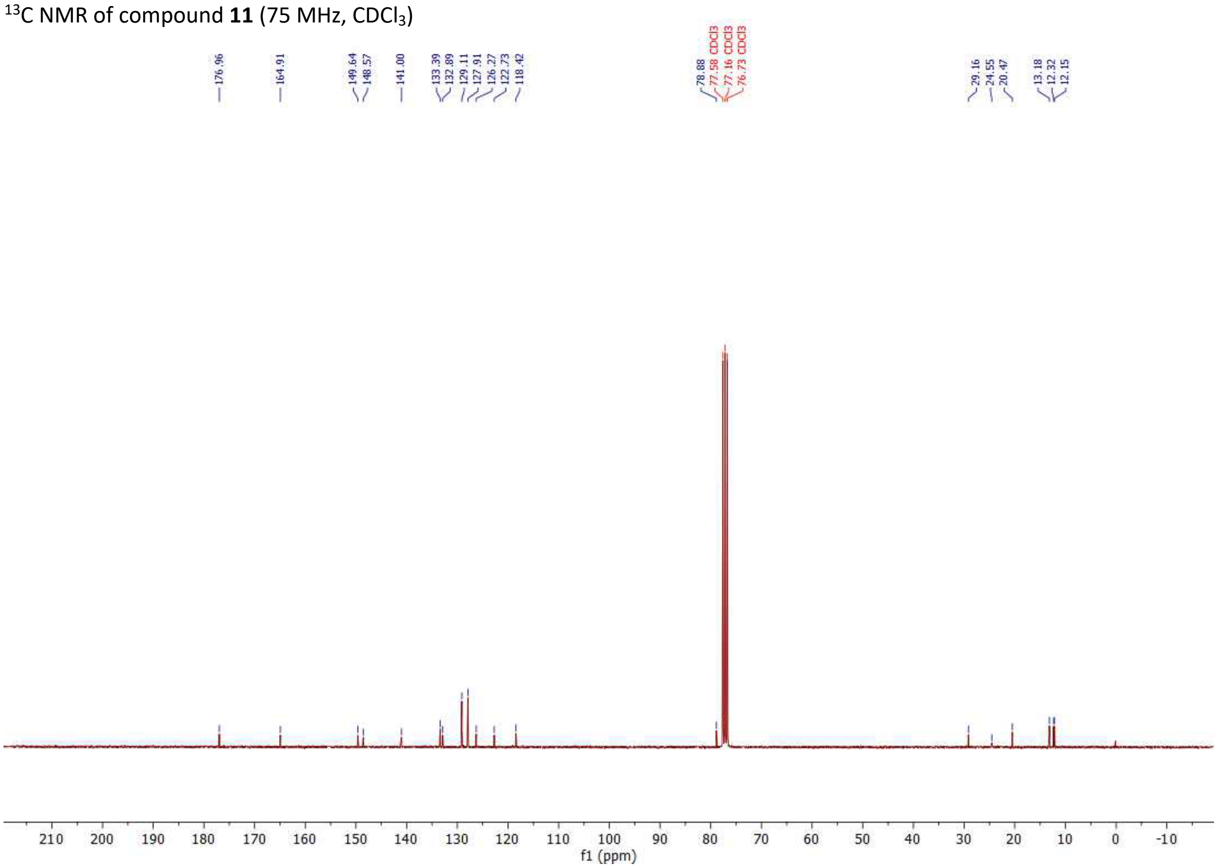

^{1}H NMR of compound **12** (300 MHz, dmso-d_6)

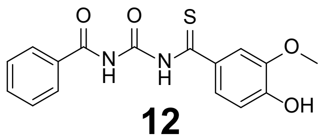

12

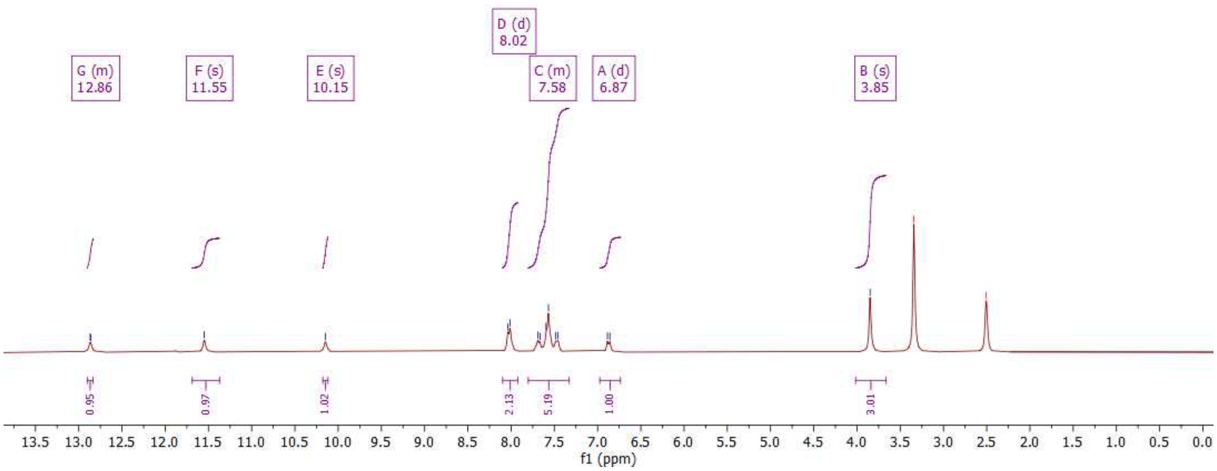

^{13}C NMR of compound **12** (75 MHz, dmso-d_6)

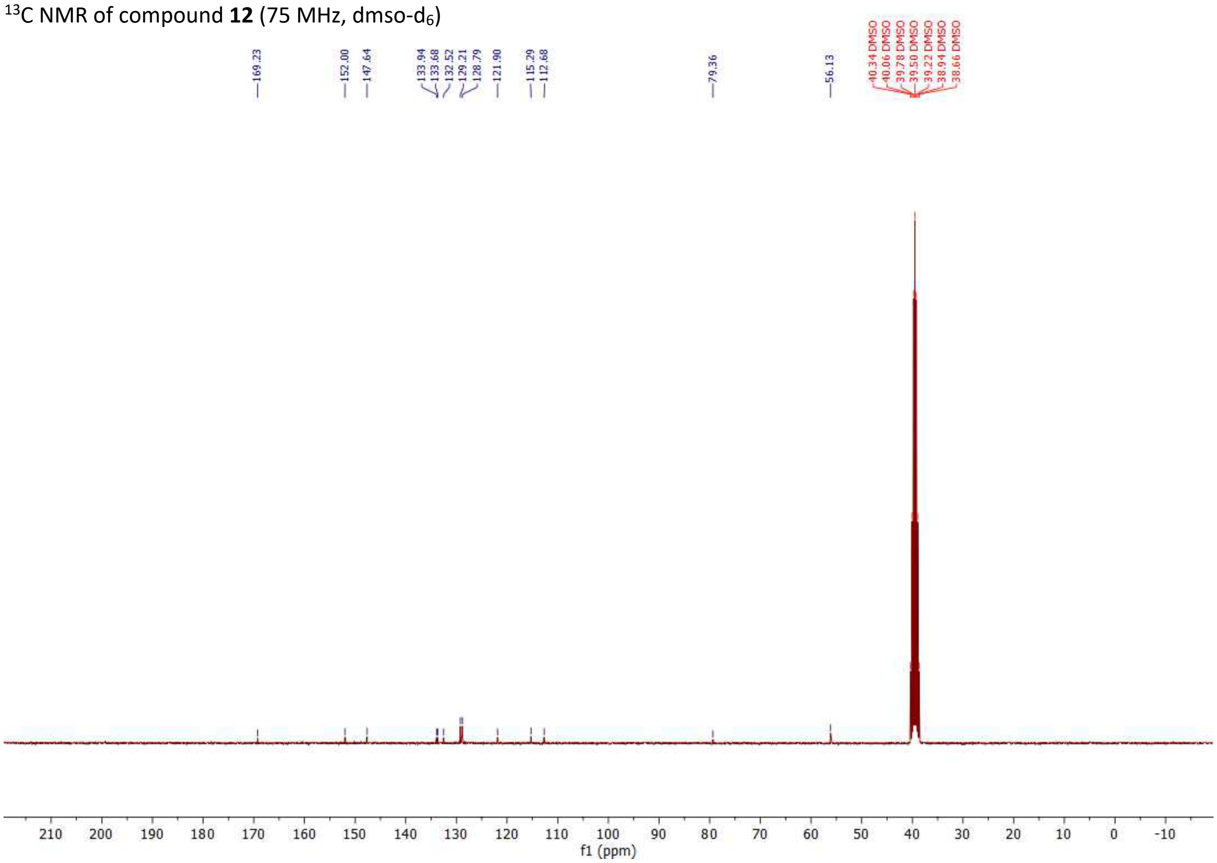

^{1}H NMR of compound **13** (300 MHz, $CDCl_3$)

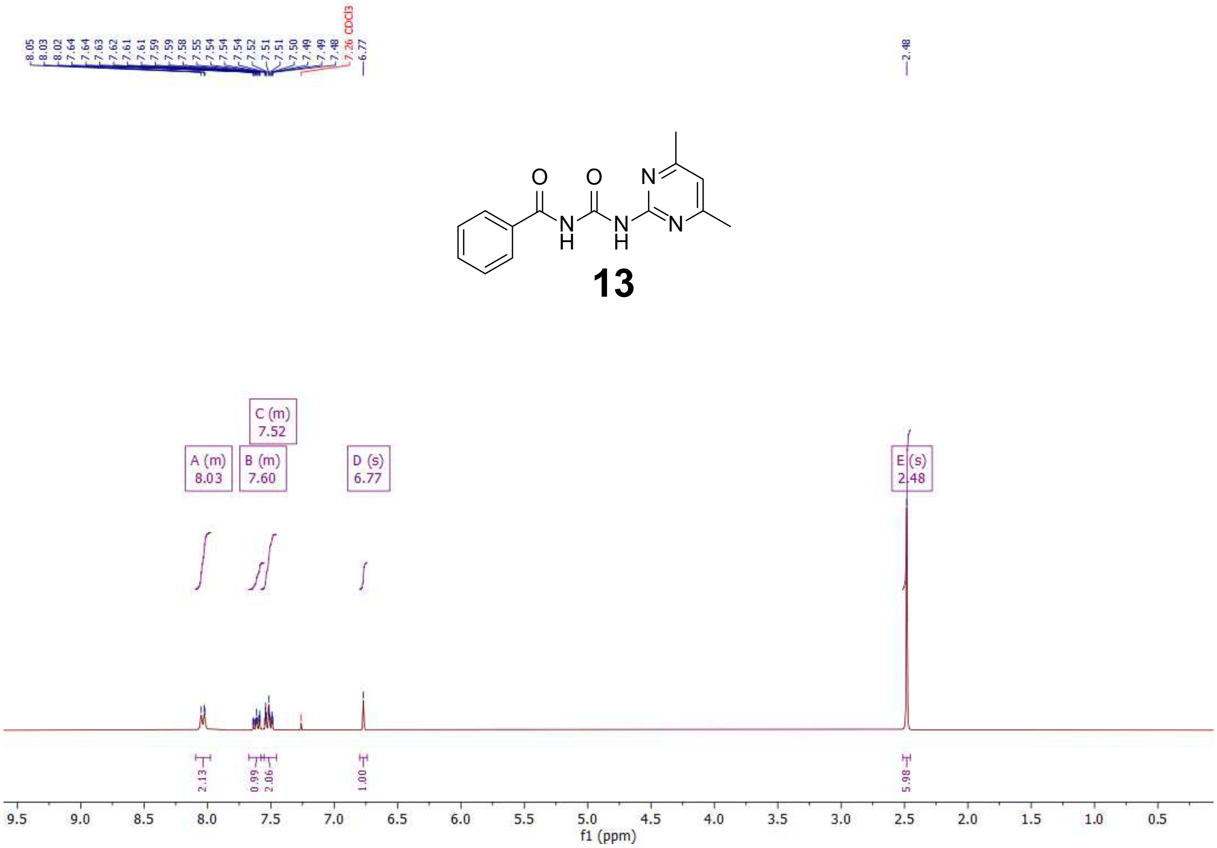

^{13}C NMR of compound **13** (75 MHz, $CDCl_3$)

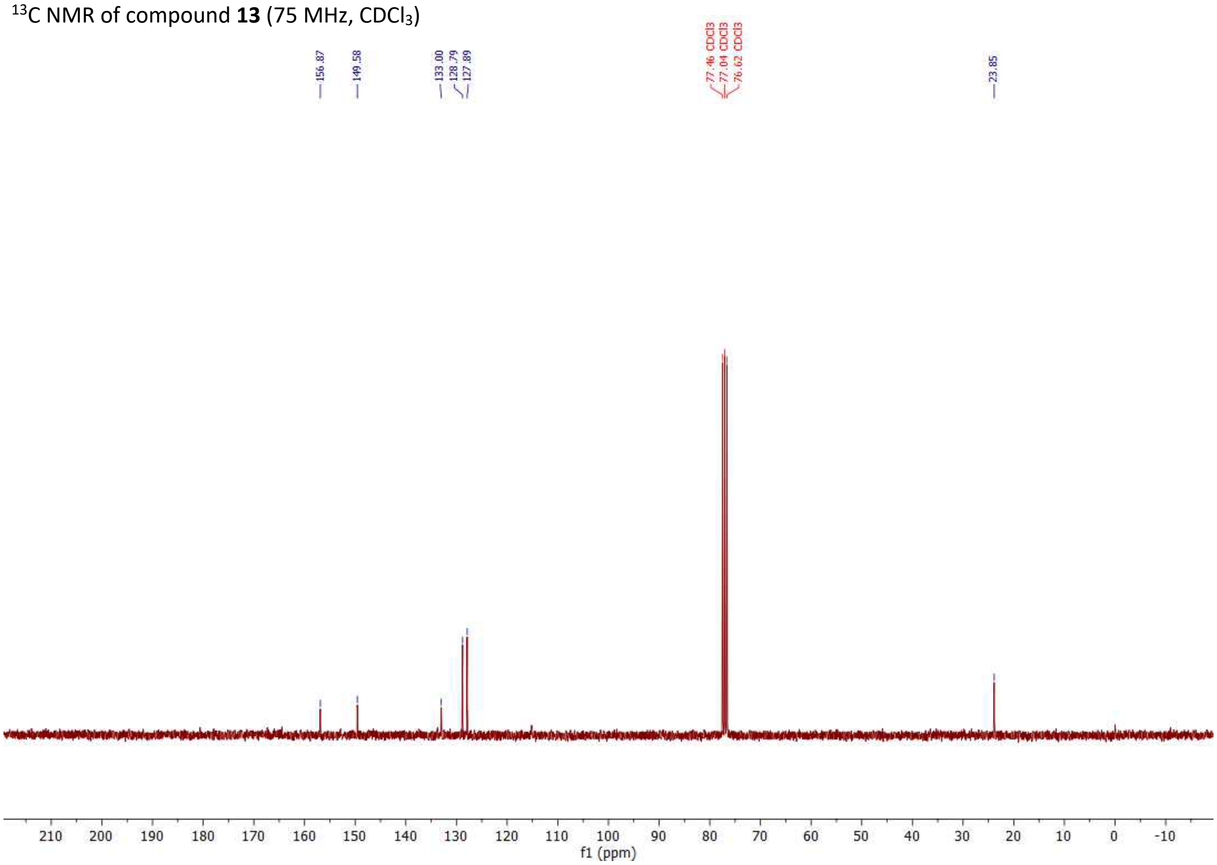

^{1}H NMR of compound **14** (300 MHz, $CDCl_3$)

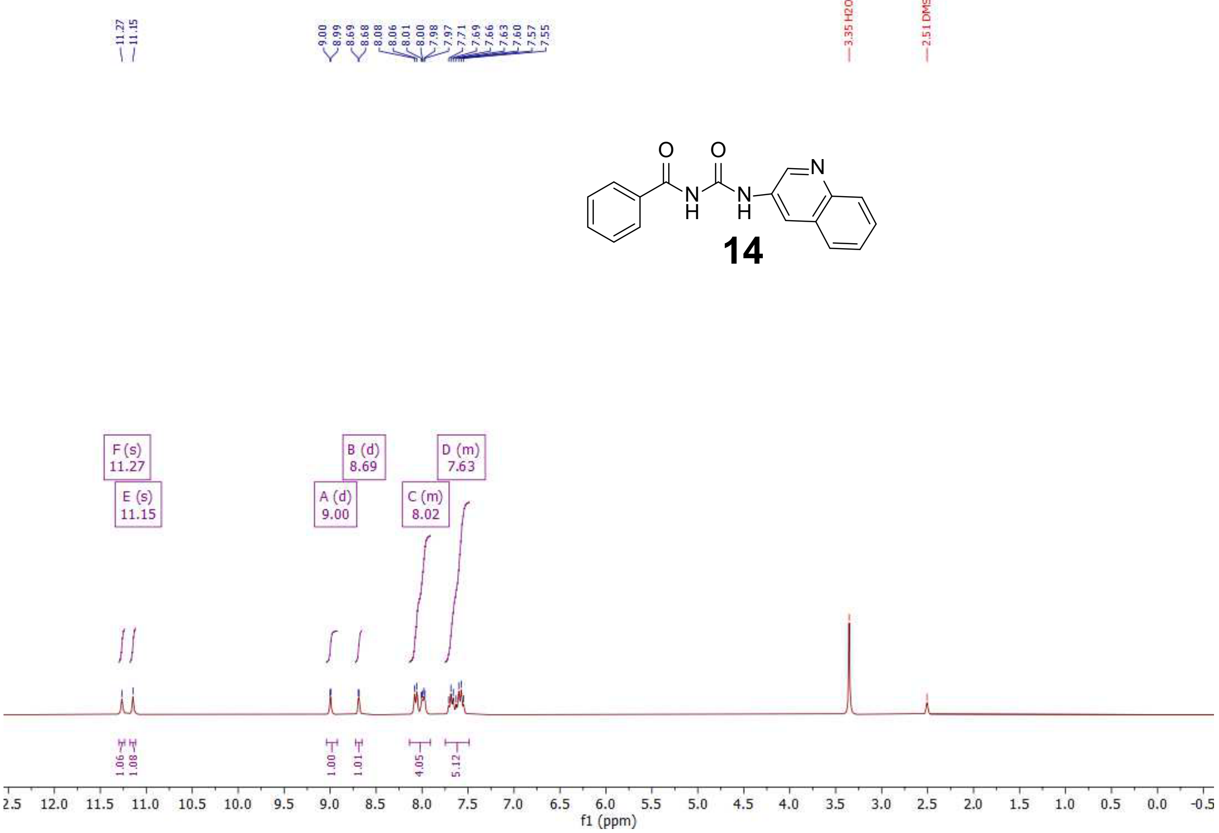

14

^{13}C NMR of compound **14** (75 MHz, $CDCl_3$)

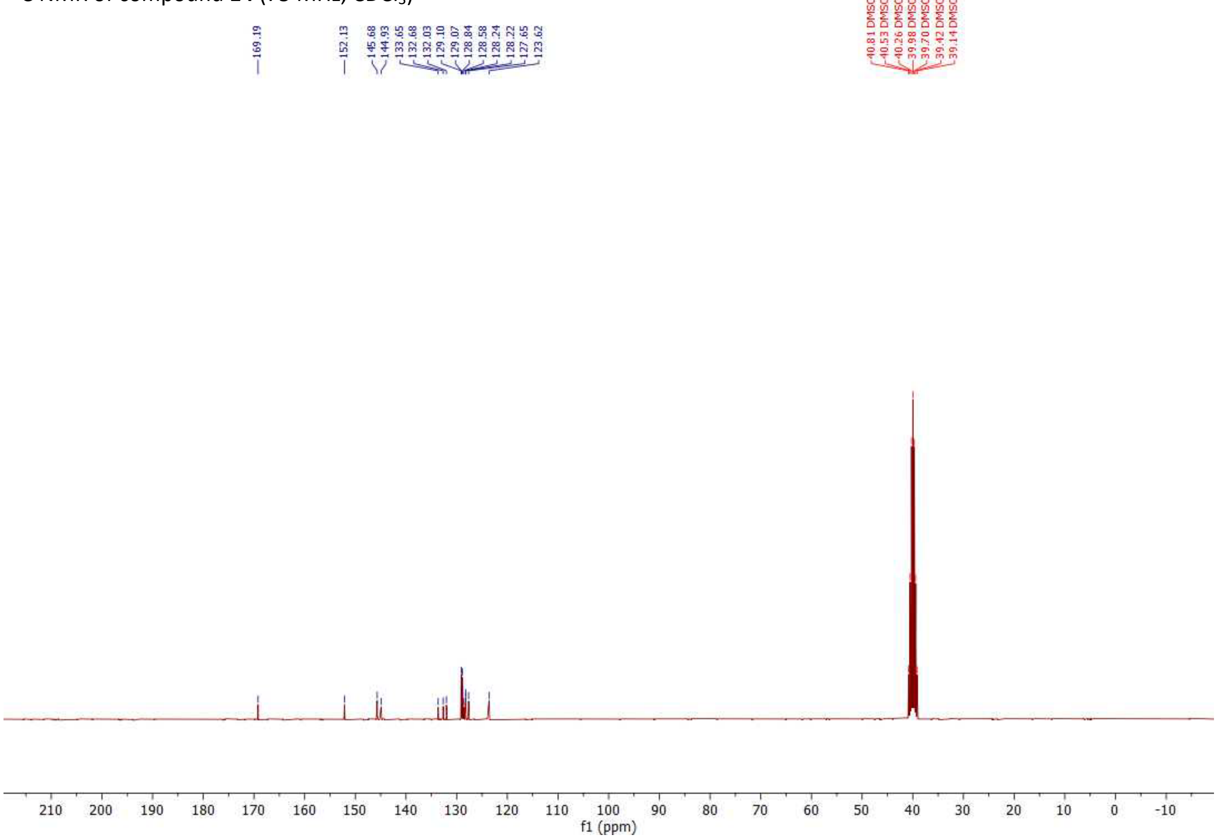

^{1}H NMR of compound **15** (75 MHz, $CDCl_3$)

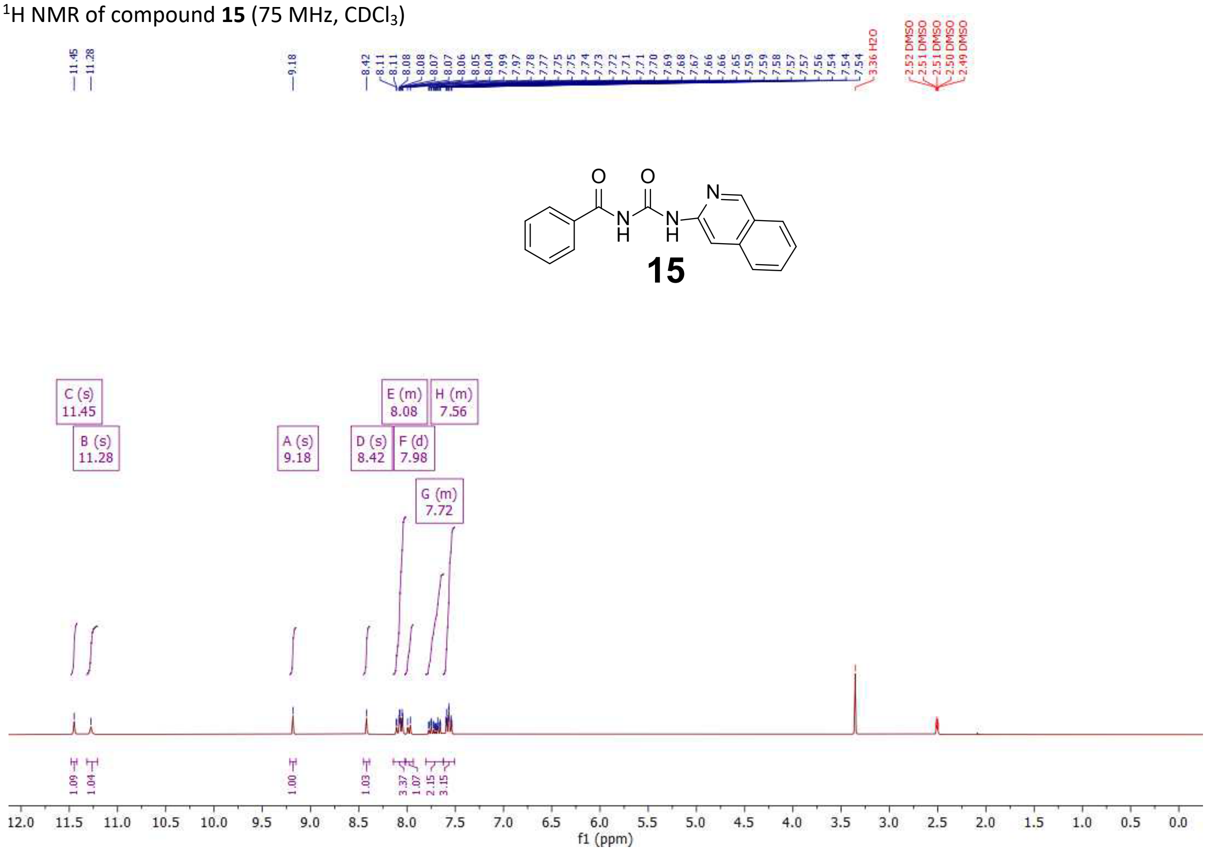

^{13}C NMR of compound **15** (75 MHz, $CDCl_3$)

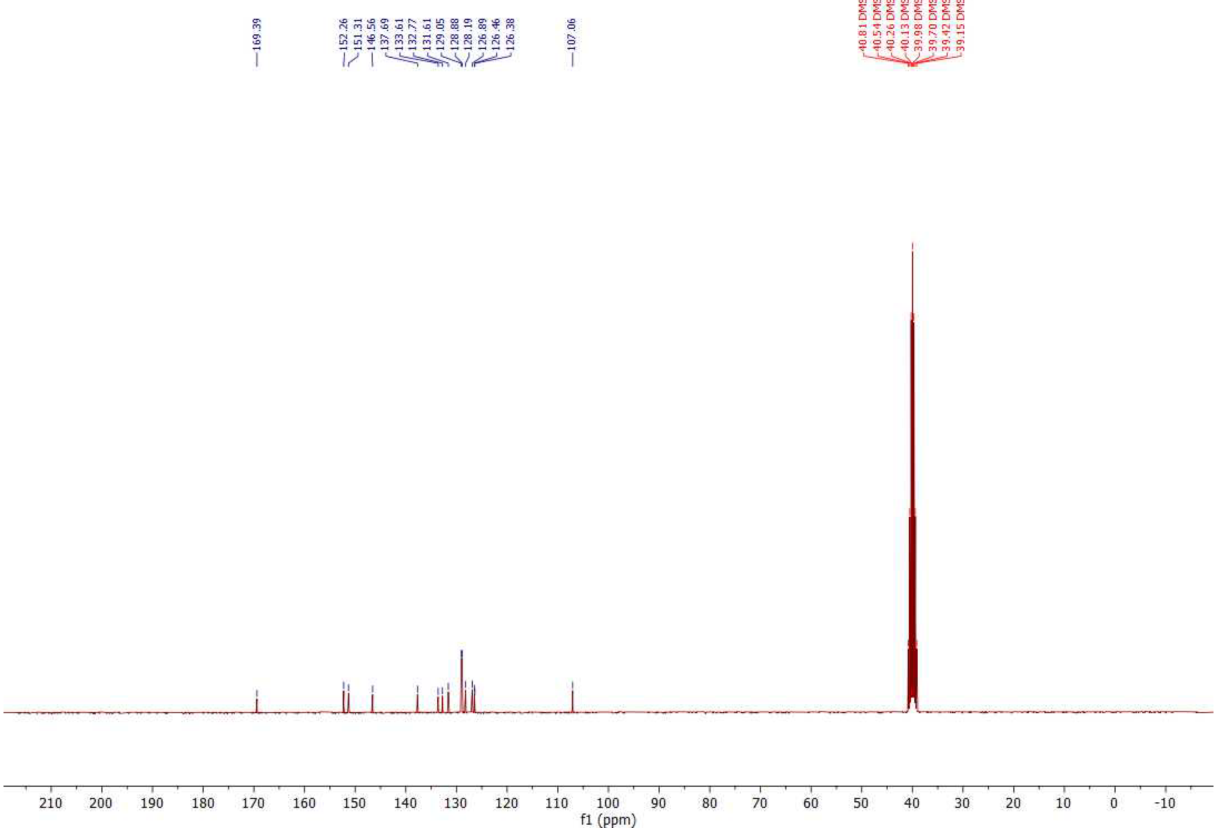

^{1}H NMR of compound **16** (75 MHz, $CDCl_3$)

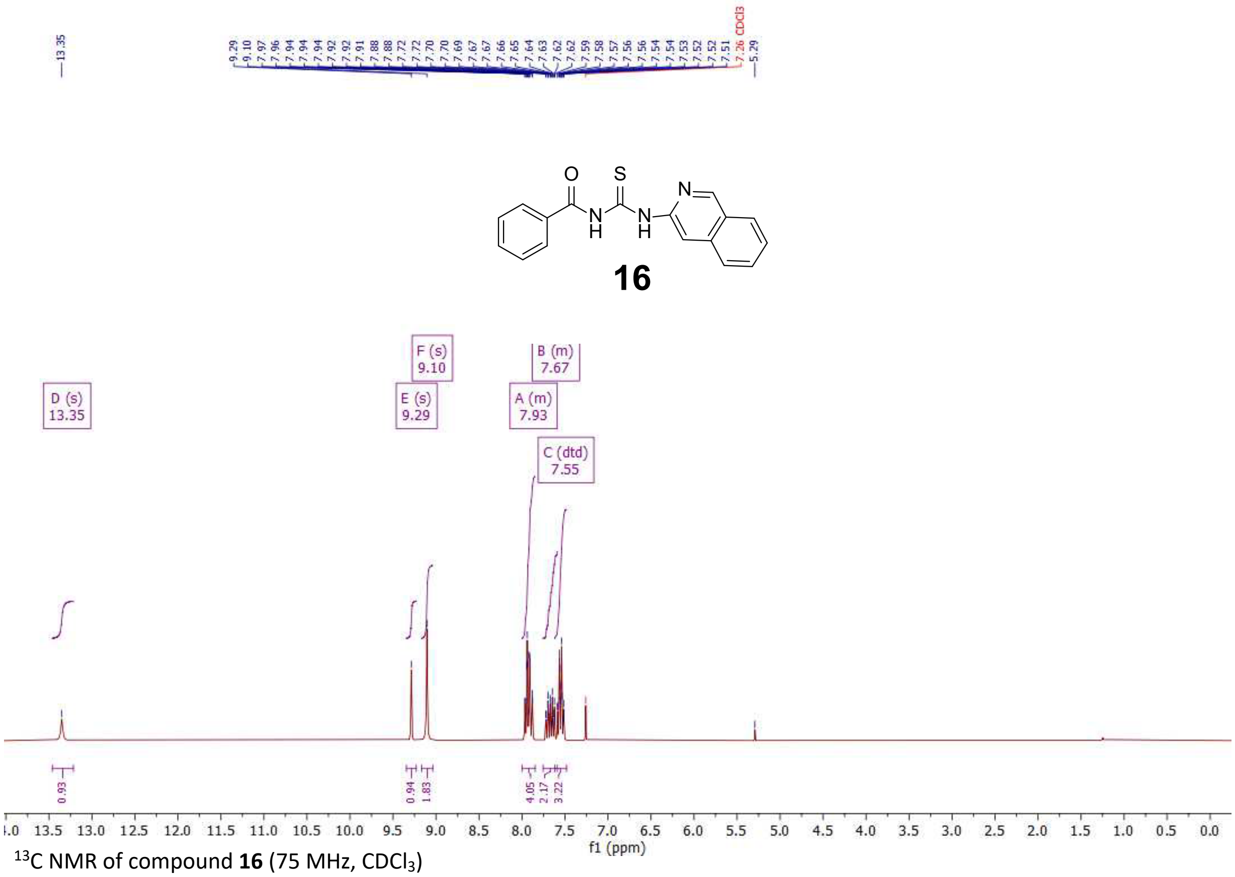

^{13}C NMR of compound **16** (75 MHz, $CDCl_3$)

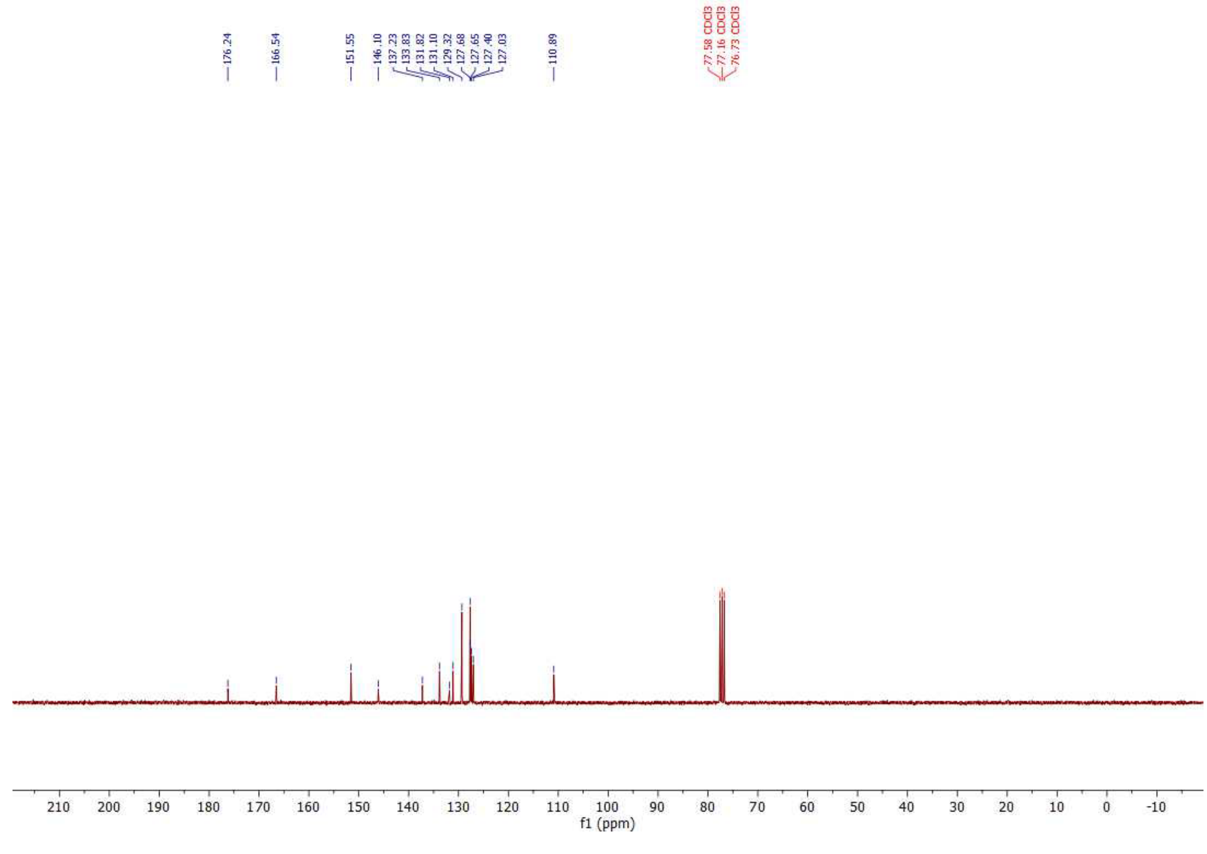

Supplement: Supplementary file 1 [file molecules-28-02319-s001.zip › molecules-2237547-supplementary.pdf]
